# Novel bivalent securinine mimetics as topoisomerase I inhibitors†
†The authors declare no competing interests.
‡
‡Electronic supplementary information (ESI) available. See DOI: 10.1039/c6md00563b


**DOI:** 10.1039/c6md00563b

**Published:** 2017-01-03

**Authors:** Wen Hou, Hui Lin, Zhen-Ya Wang, Martin G. Banwell, Ting Zeng, Ping-Hua Sun, Jing Lin, Wei-Min Chen

**Affiliations:** a College of Pharmacy , Jinan University , Guangzhou 510632 , P. R. China . Email: linjing_jnu@163.com ; Email: twmchen@jnu.edu.cn ; Fax: +86 20 8522 4766 ; Tel: +86 20 8522 1367 ; Tel: +86 20 8522 4497; b Research School of Chemistry , Institute of Advanced Studies , Australian National University , Canberra , ACT 2601 , Australia

## Abstract

A series of novel bivalent securinine mimetics incorporating different linkers between C-15 and C-15′ were synthesized and their topoisomerase I (Topo I) inhibitory activities evaluated. It was thus revealed that mimetic **R2** incorporating a rigid *m*-substituted benzene linker exhibits Topo I inhibitory activity three times that of parent securinine. Comprehensive structure–activity relationship analyses in combination with docking studies were used to rationalize the potent activity of these bivalent mimetics. Mechanistic studies served to confirm the deductions arising from docking studies that the active bivalent mimetics not only inhibited complexation between Topo I and DNA but also stabilized the Topo I–DNA complex itself.

## Abstract 2

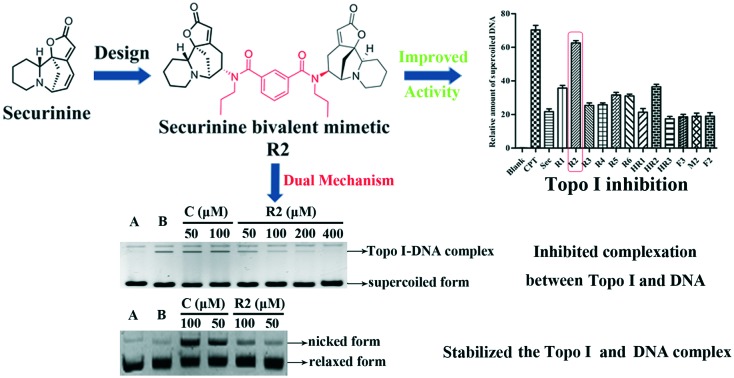
A series of novel bivalent securinine mimetics incorporating different linkers between C-15 and C-15′ were synthesized and their topoisomerase I (Topo I) inhibitory activities evaluated.

## 1 Introduction

The topoisomerase I (Topo I) enzymes are involved in the cutting of one strand of double-stranded and supercoiled DNA, relaxing that strand then re-annealing it in specific ways.^1^ Given its pivotal role in replication, Topo I was recognized as a target for the development of antitumor drugs in the 1980s.^2^ As a result of studies in the area the natural product camptothecin (CPT)^3^ was identified as a potent Topo I inhibitor and the semi-synthetic derivatives topotecan^4^ and irinotecan^5^ are now used clinically for treating, *inter alia*, ovarian, colon and lung cancers.

In previous studies^6^ we identified certain securinine derivatives that act as Topo I inhibitors. Since various linked/dimeric forms of other inhibitors, notably benzimidazoles, seem to exhibit better activity than the parent (monomeric) systems,^7^–^11^ we were prompted to construct novel, dimeric securinine derivatives for the purpose of examining their Topo I-inhibitory activities. Herein we describe the synthesis, using hetero-Michael addition chemistry,^12^ of eleven such bivalent securinine “mimetics” and the identification of one displaying three times the inhibitory activity of the parent (monomeric) system. Further, we detail comprehensive structure–activity relationship analyses and docking studies that reveal the origins of this enhanced activity. Mechanistic investigations, including DNA-cleavage studies and electrophoretic mobility shift assays (EMSAs), as well as DNA-insertion assays, are also described that confirm the inferences drawn from the docking studies.

## 2 Results and discussion

### 2.1 Chemical synthesis of bivalent securinine mimetics

**Fig. 1:**
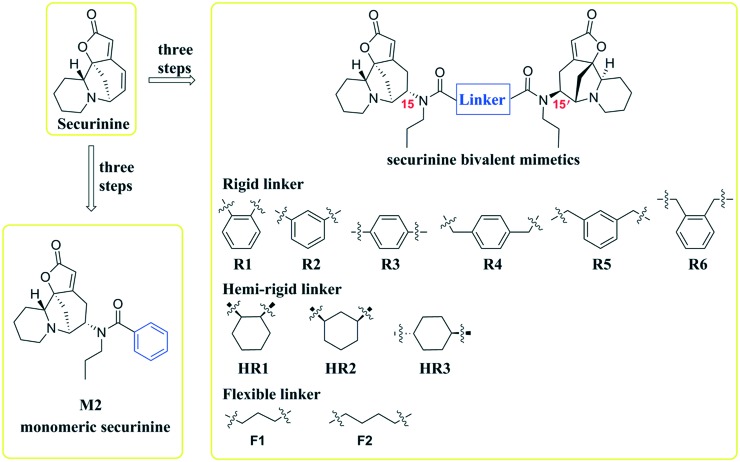
Structures of the target molecules.

Inspired by a report^11^ revealing that certain bisbenzimidazoles were more efficacious than the corresponding monomers as Topo I inhibitors, eleven compounds, namely **R1–R6**, **HR1–HR3**, **F2** and **F3** (Fig. 1), were identified as the most suitable substrates with which to investigate whether or not bivalent securinine mimetics are more active than the analogous monomeric systems. In order to establish any effects that might be exerted by the linker joining the two monomeric units in these bivalent systems, three types of mimetic were designed, namely those incorporating a rigid linker (labeled as the **R** series), those with a hemi-rigid linker (the **HR** series) and those with a flexible one (the **F** series). Additionally, a monomer **M2** (Fig. 1) embodying the same linker unit as the most active bivalent mimetic **R2** but lacking the second securinine unit was also prepared in order to study whether or not two such units (rather than just the linker itself) was a necessary requirement for enhanced activity.

**Scheme 1:**
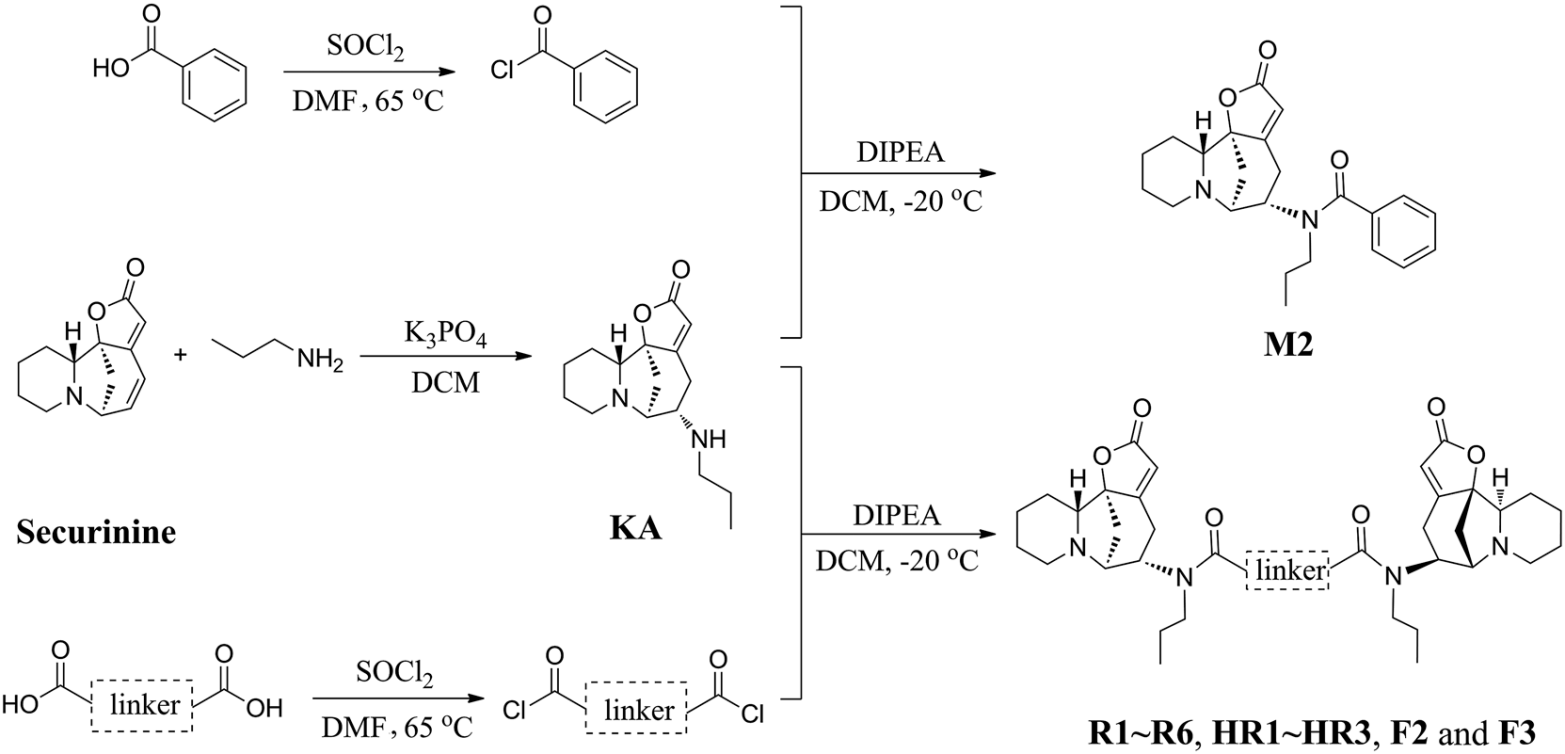
Synthetic route used to obtain the targeted securinine analogues.

The concise routes shown in Scheme 1 allowed for the synthesis of all twelve securinine derivatives and the structures of these were established using ^1^H and ^13^C NMR spectroscopic as well as low- and high-resolution electrospray ionization (ESI) mass spectrometric techniques.

### 2.2 Biological studies

#### 2.2.1 Evaluation of Topo I inhibitory properties

**Fig. 2:**
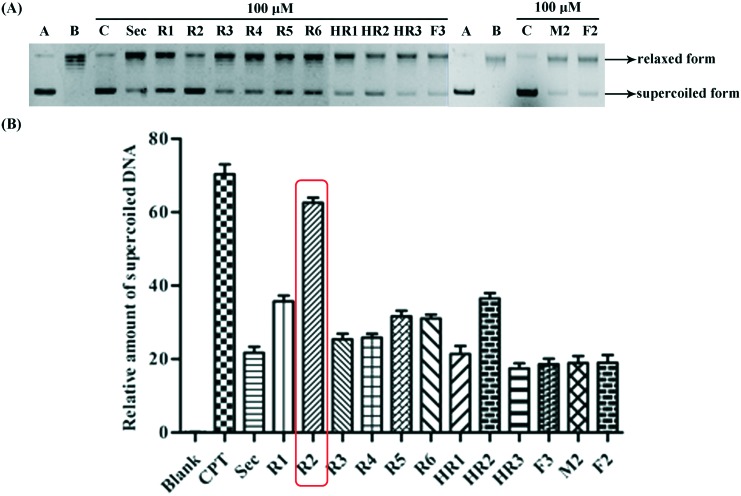
(A) Gel electrophoretic chromatogram arising from Topo I inhibitory of the title securinine derivatives. CPT used a positive control. Supercoiled pBR322 DNA was incubated with Topo I in the presence or absence of compound at 37 °C for 0.5 h. Lane A – pBR322 DNA alone. Lane B – mixture of pBR322 DNA and Topo I. Lane C – mixture of pBR322 DNA, Topo I and 100 μM CPT. Other lanes – mixtures of pBR322 DNA, Topo I and 100 μM of test compound; (B) gray scale value analysis of results shown in (A).

A Topo I inhibitory assay^13^ was employed as an initial screen to establish the efficacy (or otherwise) of the abovementioned bivalent securinine mimetics. In this assay, CPT, an established Topo I inhibitor,^3^ was used as the positive control. As shown in Fig. 2, when 100 μM solutions of securinine and each of the mimetics was tested the amount of the supercoiled form of DNA (the lower band in Fig. 2A) present at a given point in time varied significantly but was always evident and thus implying that the tested compounds were acting as inhibitors of Topo I. All of the **R** series bivalent mimetics were stronger inhibitors than securinine itself with the most effective (**R2**) being nearly as active as the positive control CPT and almost three times as active as the parent compound. These results clearly demonstrate that bivalent securinine mimetics can act as effective Topo I inhibitors. In the **HR** series only one, **HR2**, showed higher activity (but only just) than securinine, while in the **F** series no enhancement of activity (relative to securinine) was observed. This was also the case for compound **M2**. These results indicate that the presence of a second securinine unit can impart enhanced activity but that the manner in which the two units are joined is critical. Specifically, a conformationally limiting (rigid) linker is pivotal as evidenced by the reduction in activity in moving from a phenyl-based group to a cyclohexyl-based one and then to a propyl linker (compare **R2** with **HR2** and with **F2**). The length of the linker also has a significant impact on the activity. Thus, increasing the length of this unit by inserting even one more carbon reduced activity significantly (compare **R2** with **R5**). The relative disposition of the securinine units was also important as evident from the variations in activity in “moving” from *meta*- to *ortho*-disubstituted linker units (compare **R2** with **R3**) or to the *para*-disubstituted equivalent (compare **R2** with **R1**). On the basis of the data acquired so far, it would seem that a rigid linker with a total length of seven atoms and incorporating an *meta*-disubstituted aryl unit is required for the construction of bivalent securinine mimetics that act as significantly enhanced Topo I inhibitors.

#### 2.2.2 Docking studies

**Fig. 3:**
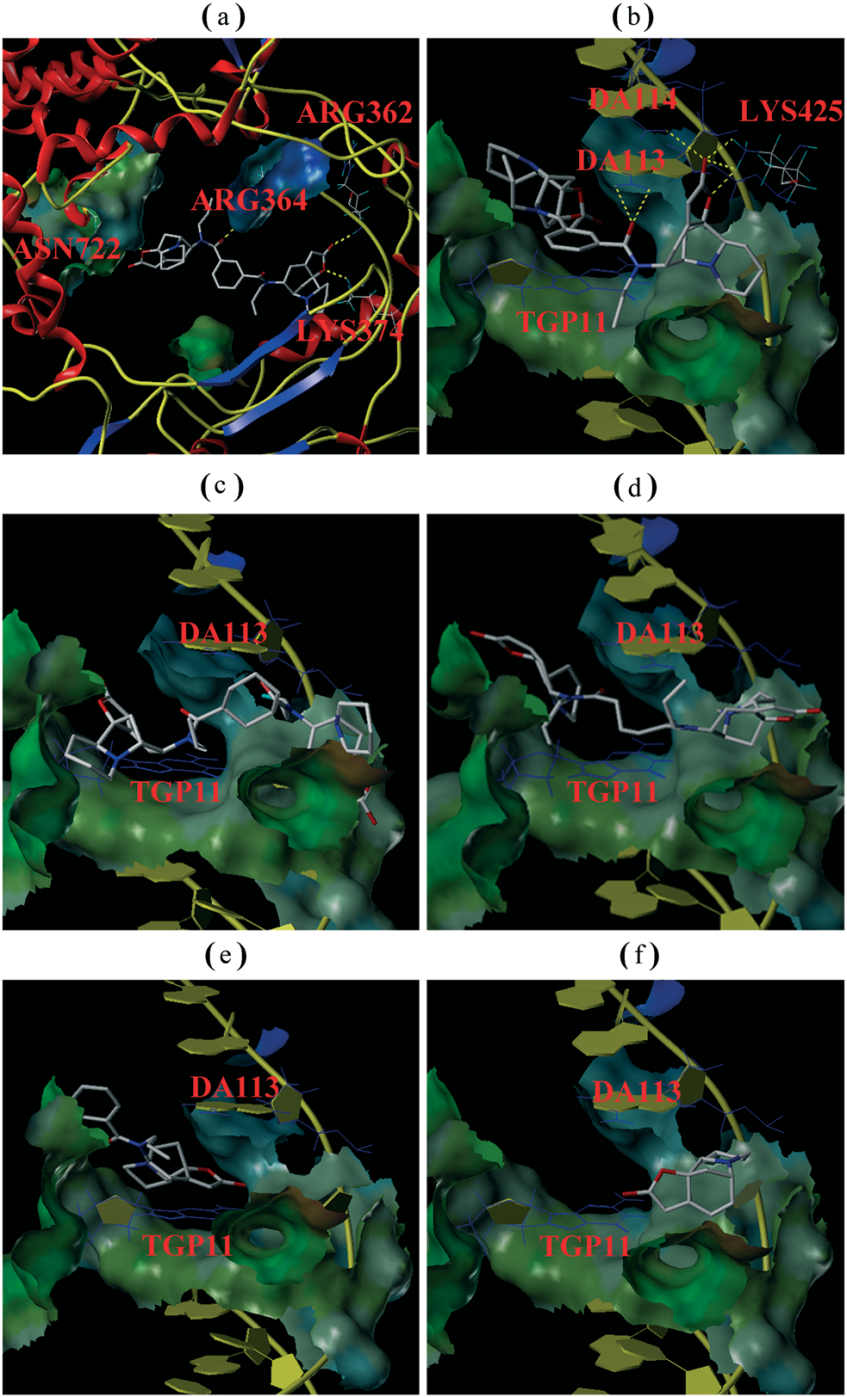
Binding model between test compounds and Topo I arising from docking studies. (a) **R2** and Topo I (no DNA); (b) **R2** and Topo I; (c) **HR2** and Topo I; (d) **F2** and Topo I; (e) **M2** and Topo I; (f) securinine and Topo I.

In order to understand the origins of structure–activity relationship profile defined above and thus determine the reasons for the excellent Topo I inhibitory activity of mimetic **R2**, docking studies were conducted on the manner in which this compound binds to the enzyme. The structure of the enzyme employed for this purpose was obtained from the Protein Data Bank (PDB ID: ; 1T8I)^14^ and complexes were generated using SYBYL-8.1 Surflex-dock. As indicated in Fig. 3a, in the absence of DNA compound **R2** binds effectively and directly to the active pocket of Topo I through the formation of five hydrogen bonds (see yellow dashed lines) with the ARG362, ARG364, LYS374 and ASN722 residues. Presumably it is this direct binding of mimetic **R2** to Topo I that prevents the normal interaction of the enzyme with DNA, an effect observed during the course of our previously reported studies of the activities of monomeric securinine derivatives.^6^ In addition, compound **R2** binds effectively to the covalent Topo I/DNA complex (Fig. 3b) through the agency of six hydrogen bonds (see yellow dashed lines) with two adjacent bases of DNA (*viz.* DA113 and DA114 – blue structures) and the LYS425 residue of Topo I. π–π Stacking between the aryl group of the linker and TGP11 (in blue) also seems likely. Such a suite of interactions suggests that the bivalent mimetic **R2** could also stabilize the Topo I/DNA convalent complex. Accordingly, we propose that the dual capacity of compound **R2** to bind directly to Topo I and to stabilize the complex of this enzyme with DNA accounts for its potent inhibitory activity. In order to investigate whether or not π–π stacking between the aryl linker within compound **R2** and the nucleoside bases of DNA is important, congeners **HR2** and **F2** lacking an aromatic residue were also subject to docking studies. As shown in Fig. 3c and d both of these failed to bind to the Topo I/DNA covalent complex and no hydrogen bonding and π–π stacking interactions were observed. This strongly suggests, therefore, that π–π stacking between the aromatic residues of the linker associated with bivalent mimetic **R2** and the nucleotide bases of DNA has a major (beneficial) impact on activity.

In order to determine whether or not both securinine units within the bivalent mimetics are necessary for effective binding, the monomeric congener **M2** (possessing the same linker as the most active compound **R2** but lacking a second securinine residue) and the parent system (*viz.* securinine) were each subject to docking analysis. As revealed in Fig. 3e and f, neither of these reference substrates bind effectively with the Topo I/DNA complex and thus highlighting the importance of the presence of a bivalent motif.

Overall, then, these docking studies strongly suggest that the excellent Topo I inhibitory activity of the bivalent mimetic **R2** arises through a dual inhibitory mechanism involving, (i), binding of it, through π–π stacking, with the covalent Topo I/DNA complex (and thereby stabilizing the same) and, (ii), binding of the second securinine unit within this inhibitor with Topo I (and so inhibiting the normal mode of action of the enzyme).

#### 2.2.3 Electrophoretic mobility shift assays (EMSAs)

**Fig. 4:**
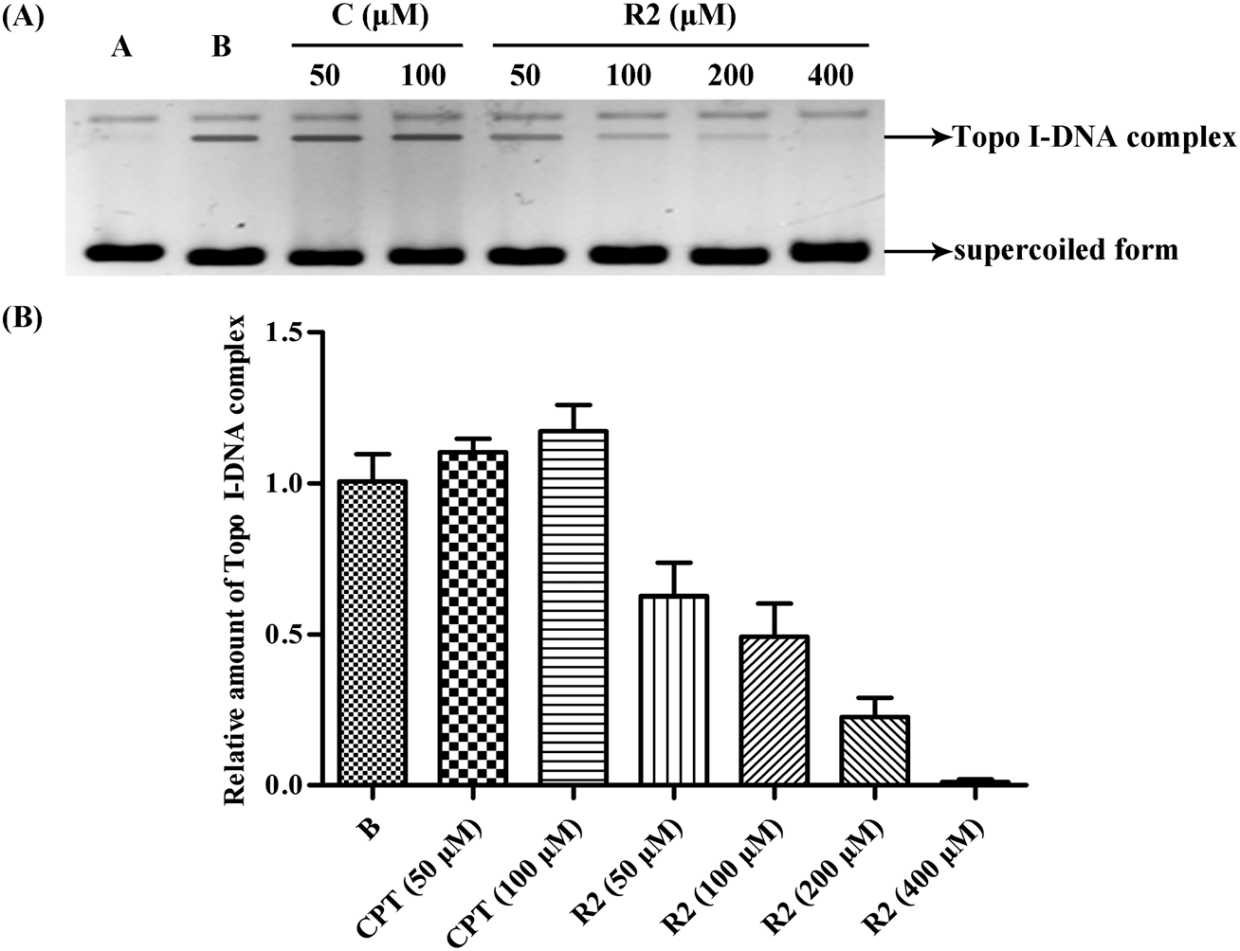
(A) Gel electrophoretic chromatogram arising from EMSA of compound **R2**. CPT used as a negative control. Lane A – pBR322 DNA only. Lane B – mixture of pBR322 DNA and Topo I. Line C – mixture of pBR322 DNA, Topo I and 50 or 100 μM CPT. Other lanes – mixture of pBR322 DNA, Topo I and 50, 100, 200, or 400 μM **R2**; (B) gray scale value analysis of results shown in (A).

In order to substantiate the hypotheses arising from our docking studies, an electrophoretic mobility shift assay (EMSA)^15^ was carried out so as to establish whether or not compound **R2** inhibits the binding of DNA with Topo I. As shown in Fig. 4, upon addition of successive aliquots of compound **R2** to the Topo I/DNA complex the concentration of the latter decreased linearly. This result clearly demonstrates that the bivalent mimetic **R2** blocks (inhibits) the complexation of Topo I with DNA and so suggesting a high affinity of compound **R2** for Topo I as depicted in Fig. 3a.

#### 2.2.4 DNA-cleavage assay

**Fig. 5:**
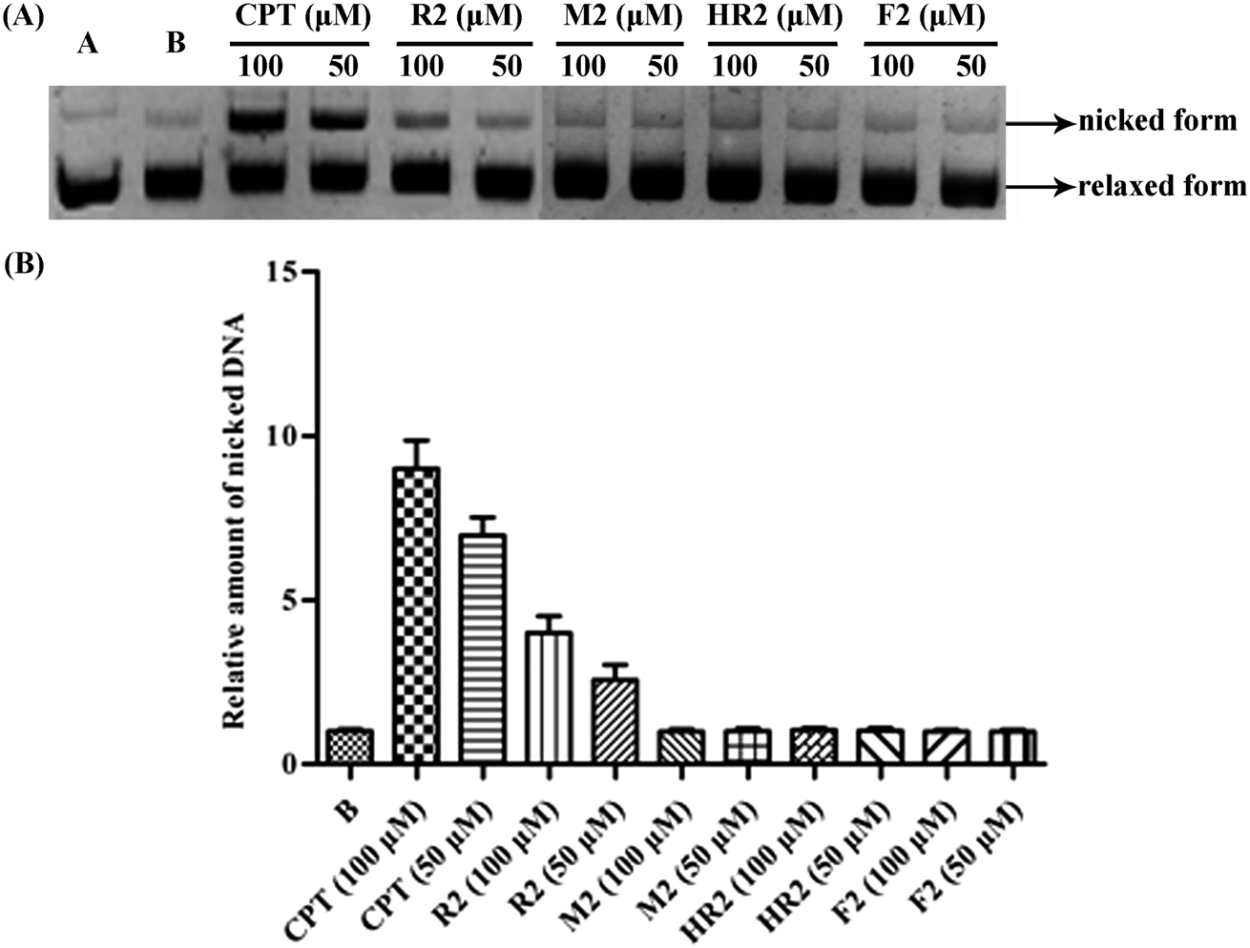
(A) Gel electrophoretic chromatogram arising from Topo I-mediated assay of compounds **R2**, **M2**, **HR2**, and **F2**. CPT used as a positive control. Lane A – pBR322 DNA only. Lane B – mixture of pBR322 DNA and Topo I. Other lanes – mixture of pBR322 DNA, Topo I and 50, 100 μM of test compound; (B) gray scale value analysis of A.

A DNA-cleavage assay was also conducted in order to establish whether or not compound **R2** stabilizes the Topo I/DNA complex as predicted by the above-mentioned docking studies. CPT served as a positive control in this assay and on employing it (Fig. 5) at increasing concentrations the quantities of nicked DNA (see upper bands) accumulated in a linear manner, an outcome consistent with earlier reports.^16^,^17^ Nicked DNA bands were also observed with compound **R2**, especially at 100 μM concentrations, although these were not as conspicuous as the ones observed with CPT (an outcome consistent with the dual binding mode of **R2** suggested by the docking study). These cleavage assays therefore also support the proposition that mimetic **R2** binds to and thus stabilizes the Topo I/DNA covalent complex. In contrast, and as predicted by the docking studies, compounds **HR2**, **F2** and **M2**, failed to generate nicked DNA in the same assay.

#### 2.2.5 DNA-intercalation assay

**Fig. 6:**
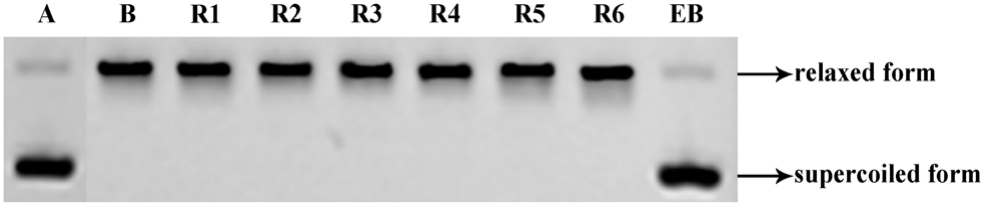
DNA-insertion assay of the **R** series compounds. EB used as a positive control. Supercoiled pBR322 DNA was incubated at 37 °C for 0.5 h with Topo I in the presence or absence of EB, and with the test compounds. Lane A – pBR322 DNA alone. Lane B – mixture of pBR322 DNA and Topo I. Lane EB – mixture of pBR322 DNA, Topo I and 25 μM EB. Other lanes – mixture of pBR322 DNA, Topo I and 100 μM of test compound.

It has been reported^18^ that Topo I inhibitors can exert their effects by one (or more) of three possible mechanisms, namely through DNA intercalation (as seen with ethidium bromide or EB), through retarding formation of the normal Topo I/DNA complex or through stabilizing this complex. In order to establish whether or not the mimetics described here can act by the first pathway, a DNA-insertion assay was carried out on the **R**-series compounds and EB was used as the positive control. As shown in Fig. 6, on treating Topo I with 25 μM EB the activity of the enzyme was shutdown. In contrast, in the same assay Topo I remained able to convert supercoiled DNA (lower bands in Fig. 6) into a relaxed form (upper bands) in the presence of the **R**-series mimetics. Accordingly, and given the outcomes of the other assays detailed above, we propose that the more potent bivalent mimetics, such as compound **R2**, act by the second and third inhibitory mechanisms but not by the first.

## 3 Conclusions

In this study, a series of novel securinine bivalent mimetics and one monomer were designed and synthesized for evaluation as Topo I inhibitors. Compound **R2** was found to display about three times the Topo I inhibitory activity of securinine itself and so demonstrating that the development of bivalent securinine mimetics is a novel and feasible strategy for constructing effective inhibitors of this crucial enzyme. Furthermore, through the combined application of docking studies and mechanistically relevant assays, the two-fold binding mode and consequent dual effects of this potent and structurally novel^19^ inhibitor (*viz.***R2**) have been revealed. The structure–activity relationship data and docking studies reported here also provide a wealth of information for the further development of bivalent mimetics as potentially valuable new Topo I inhibitors.

## 4 Experimental

### 4.1 Chemistry

#### General procedures

Unless otherwise specified, proton (^1^H) and carbon (^13^C) NMR spectra were recorded at 18 °C in base-filtered CDCl_3_ on a Varian spectrometer operating at 300 MHz for proton and 75 MHz for carbon nuclei. For ^1^H NMR spectra, signals arising from the residual protio-forms of the solvent were used as the internal standards. ^1^H NMR data are presented as follows: chemical shift (*δ*) [multiplicity, coupling constant(s) *J* (Hz), relative integral] where multiplicity is defined as: s = singlet; d = doublet; t = triplet; q = quartet; m = multiplet or combinations of the above. The signal due to residual CHCl_3_ appearing at *δ*_H_ 7.26 and the central resonance of the CDCl_3_ “triplet” appearing at *δ*_C_ 77.1(6) were used to reference ^1^H and ^13^C NMR spectra, respectively. Low-resolution ESI mass spectra were recorded on a Finnigan LCQ Advantage MAX mass spectrometer and fitted with a 4000 Q TRAP or Agilent 6130 Quadruple LC/MS. High-resolution mass spectra (HR-MS) were obtained on an Agilent 6210 series LC/MSD TOF mass spectrometer. Analytical thin layer chromatography (TLC) was performed on aluminium-backed 0.2 mm thick silica gel 60 F_254_ plates as supplied by Merck. Eluted plates were visualized using a 254 nm UV lamp and/or by treatment with a suitable dip followed by heating. Flash chromatographic separations were carried out following protocols defined by Still *et al.*^20^ with silica gel 60 (40–63 μm) as the stationary phase and using the AR- or HPLC-grade solvents indicated. Starting materials and reagents were generally available from the Sigma-Aldrich, Merck, TCI, Strem or Lancaster Chemical Companies and were used as supplied. Drying agents and other inorganic salts were purchased from the AJAX, BDH or Unilab Chemical Companies. Where necessary, reactions were performed under an inert atmosphere. Compounds **F2** and **F3** were obtained using previously reported methods.^21^

#### Specific chemical transformations

##### (5*S*,6*S*,11a*S*,11b*S*)-5-(Propylamino)-5,6,9,10,11,11a-hexahydro-8*H*-6,11b-methanofuro[2,3-*c*]pyrido[1,2-*a*]azepin-2(4*H*)-one (**KA**)

The title compound was prepared using a modification of a previously reported procedure.^21^ Thus, a magnetically stirred solution of securinine (1.90 g, 9.0 mmol, 1.0 equiv.) in dichloromethane (DCM, 30 mL) maintained at ambient temperatures was treated with K_3_PO_4_ (9.5 mg, 0.045 mmol, 0.05 equiv.) then *n*-propylamine (1.59 g, 27.0 mmol, 3.0 molar equiv.). After 8 h the now milk-white mixture was concentrated under reduced pressure and the residue diluted with water (100 mL) then extracted with DCM (3 × 50 mL). The combined organic phases were dried (MgSO_4_), filtered and concentrated under reduced pressure. The residue thus obtained was subject to flash chromatography (silica gel, 200 : 1 v/v DCM/MeOH) to give, after concentration of the appropriate fractions, compound **KA**^21^ (1.20 g, 49%) as a pale-yellow oil. ^1^H NMR (300 MHz, CDCl_3_) *δ* 5.57 (d, *J* = 2.1 Hz, 1H), 3.22–3.16 (m, 1H), 3.01 (dd, *J* = 6.3 and 4.2 Hz, 1H), 2.92–2.75 (m, 4H), 2.58–2.40 (m, 4H), 1.89–1.79 (m, 2H), 1.59–1.25 (m, 8H), 0.85 (t, *J* = 7.5 Hz, 3H); ^13^C NMR (75 MHz, CDCl_3_) *δ* 174.5, 173.2, 110.9, 91.4, 63.0, 59.8, 59.6, 49.7, 48.8, 32.3, 30.4, 25.7, 23.8, 23.3, 21.5, 11.9. These data were essentially identical, in all respects, with those reported^21^ previously.

##### *N*-((5*S*,6*S*,11a*S*,11b*S*)-2-Oxo-2,4,5,6,9,10,11,11a-octahydro-8*H*-6,11b-methanofuro[2,3-*c*]pyrido[1,2-*a*]azepin-5-yl)-*N*-propylbenzamide (**M2**)

Benzoic acid (10 mmol) was added to thionyl chloride (5 mL, 25 mmol) and the resulting solution heated, with magnetic stirring, under reflux for 5 h while being maintained under an atmosphere of nitrogen. The cooled reaction mixture was concentrated under reduced pressure and a portion of the benzoyl chloride (0.3 mmol, 0.6 equiv.) thus obtained added, dropwise over 0.33 h, to a magnetically stirred solution of **KA** (138 mg, 0.5 mmol, 1.0 equiv.) in anhydrous DCM (10 mL) containing di-iso-propylethylamine (DIPEA, 387 mg, 1.5 mmol, 3.0 equiv.) maintained at –20 °C. After 1 h the reaction mixture was warmed to *ca.* 22 °C, stirred at this temperature for 16 h then treated with aqueous NaHCO_3_ so as to achieve pH ≥ 8. The ensuing mixture was concentrated under reduced pressure and the residue extracted with DCM (3 × 20 mL). The combined organic phases were dried (MgSO_4_), filtered and concentrated under reduced pressure and the residue subjected to flash chromatography (silica gel, 100 : 1 v/v DCM : MeOH) to give, after concentration of the appropriate fractions, compound **M2** (61 mg, 32%) as a brown solid. ^1^H NMR (300 MHz, CDCl_3_) *δ* 7.40–7.34 (m, 3H), 7.32–7.26 (m, 2H), 5.67 (s, 1H), 4.24 (t, *J* = 15.5 Hz, 1H), 3.27 (dd, *J* = 15.5 and 7.5 Hz, 1H), 3.15 (m, 1H), 2.90 (m, 5H), 2.59 (dd, *J* = 11.0 and 5.8 Hz, 1H), 1.92–1.72 (m, 2H), 1.63–1.44 (m, 3H), 1.42–1.21 (m, 5H), 0.70 (t, *J* = 6.4 Hz, 3H); ^13^C NMR (75 MHz, CDCl_3_) *δ* 172.8, 172.5, 165.1, 137.3, 129.4, 128.6, 126.2, 110.3, 90.9, 63.0, 60.0, 57.6, 48.3, 33.6, 29.7, 27.3, 26.4, 24.2, 24.0, 22.1, 11.2; MS (ESI, +ve) *m*/*z* 381 [(M + H)^+^, 100%]; HRMS (ESI, +ve) *m*/*z* (M + H)^+^ calcd for C_23_H_29_N_2_O_3_ 381.2173, found 381.2174.

#### Generalised procedure for the preparation of the bivalent securinine mimetics **R1–R6** and **HR1–HR3**

The relevant diacid (10 mmol) associated with the linker unit of the title compounds was added to thionyl chloride (5 mL, 25 mmol) and the resulting solution heated, with magnetic stirring, under reflux for 5 h while being maintained under an atmosphere of nitrogen. The cooled reaction mixture was concentrated under reduced pressure and a portion of the diacid dichloride (0.3 mmol, 0.6 equiv.) thus obtained was added, dropwise over 0.33 h, to a magnetically stirred solution of **KA** (138 mg, 0.5 mmol, 1.0 equiv.) in anhydrous DCM (10 mL) containing DIPEA (387 mg, 1.5 mmol, 3.0 equiv.) maintained at –20 °C. After 1 h the reaction mixture was warmed to *ca.* 22 °C, stirred at this temperature for 16 h then treated with anhydrous NaHCO_3_ so as to achieve pH ≥ 8. The ensuing mixture was concentrated under reduced pressure and the residue extracted with DCM (3 × 20 mL). The combined organic phases were dried (MgSO_4_), filtered and concentrated under reduced pressure and the residue subjected to flash chromatography (silica gel, 100 : 1 v/v DCM : MeOH) to give the relevant diamide. The spectral data sets acquired on each of these are given below.

##### *N* ^1^-((5*S*,6*S*,11a*R*,11b*S*)-2-Oxo-2,4,5,6,9,10,11,11a-octahydro-8*H*-6,11b-methanofuro[2,3-*c*]pyrido[1,2-*a*]azepin-5-yl)-*N*^2^-((5*S*,6*S*,11a*S*,11b*S*)-2-oxo-2,4,5,6,9,10,11,11a-octahydro-8*H*-6,11b-methanofuro[2,3-*c*]pyrido[1,2-*a*]azepin-5-yl)-*N*^1^,*N*^2^-dipropylphthalamide (**R1**)

*N* ^1^-((5*S*,6*S*,11a*R*,11b*S*)-2-Oxo-2,4,5,6,9,10,11,11a-octahydro-8*H*-6,11b-methanofuro[2,3-*c*]pyrido[1,2-*a*]azepin-5-yl)-*N*^2^-((5*S*,6*S*,11a*S*,11b*S*)-2-oxo-2,4,5,6,9,10,11,11a-octahydro-8*H*-6,11b-methanofuro[2,3-*c*]pyrido[1,2-*a*]azepin-5-yl)-*N*^1^,*N*^2^-dipropylphthalamide (**R1**) was obtained as a yellow solid. Yield 33%. ^1^H NMR (300 MHz, CDCl_3_) *δ* 7.40 (broad s, 2H), 7.26 (broad s, 2H), 5.67 (broad s, 2H), 4.00–2.00 (m, 15H), 1.98–1.08 (m, 20H), 0.99–0.79 (m, 4H), 0.64 (broad s, 5H); ^13^C NMR (75 MHz, CDCl_3_) *δ* 173.8, 172.7, 171.4, 138.2, 126.5, 110.2, 90.8, 62.6, 59.9, 57.8, 53.7, 48.4, 33.6, 29.6, 27.3, 26.3, 24.1, 22.1, 11.1; MS (ESI, +ve) *m*/*z* 683 [(M + H)^+^, 100%]; HRMS (ESI, +ve) *m*/*z* [M + H]^+^ calcd for C_40_H_51_N_4_O_6_ 683.3803, found 683.3806.

*N* ^1^-((5*S*,6*S*,11a*R*,11b*S*)-2-Oxo-2,4,5,6,9,10,11,11a-octahydro-8*H*-6,11b-methanofuro[2,3-*c*]pyrido[1,2-*a*]azepin-5-yl)-*N*^3^-((5*S*,6*S*,11a*S*,11b*S*)-2-oxo-2,4,5,6,9,10,11,11a-octahydro-8*H*-6,11b-methanofuro[2,3-*c*]pyrido[1,2-*a*]azepin-5-yl)-*N*^1^,*N*^3^-dipropylisophthalamide (**R2**) was obtained a white solid. Yield 46%. ^1^H NMR (300 MHz, CDCl_3_) *δ* 7.50–7.20 (m, 4H), 5.68 (s, 2H), 4.20 (broad s, 2H), 3.50–2.70 (m, 14H), 2.61 (m, 2H), 1.90–1.76 (m, 4H), 1.59–1.16 (m, 16H), 0.70 (broad s, 6H); ^13^C NMR (75 MHz, CDCl_3_) *δ* (major rotamer) 173.7, 172.7, 171.3, 137.8, 128.7, 126.9, 124.2, 110.2, 90.8, 62.8, 59.9, 57.8, 48.3, 33.6, 29.7, 27.3, 26.3, 24.1, 22.0, 14.0, 11.2; MS (ESI, +ve) *m*/*z* 683 [(M + H)^+^, 100%]; HRMS (ESI, +ve) *m*/*z* [M + H]^+^ calcd for C_40_H_51_N_4_O_6_ 683.3803, found 683.3810.

##### *N* ^1^-((5*S*,6*S*,11a*R*,11b*S*)-2-Oxo-2,4,5,6,9,10,11,11a-octahydro-8*H*-6,11b-methanofuro[2,3-*c*]pyrido[1,2-*a*]azepin-5-yl)-*N*^4^-((5*S*,6*S*,11a*S*,11b*S*)-2-oxo-2,4,5,6,9,10,11,11a-octahydro-8*H*-6,11b-methanofuro[2,3-*c*]pyrido[1,2-*a*]azepin-5-yl)-*N*^1^,*N*^4^-dipropylterephthalamide (**R3**)

*N* ^1^-((5*S*,6*S*,11a*R*,11b*S*)-2-Oxo-2,4,5,6,9,10,11,11a-octahydro-8*H*-6,11b-methanofuro[2,3-*c*]pyrido[1,2-*a*]azepin-5-yl)-*N*^4^-((5*S*,6*S*,11a*S*,11b*S*)-2-oxo-2,4,5,6,9,10,11,11a-octahydro-8*H*-6,11b-methanofuro[2,3-*c*]pyrido[1,2-*a*]azepin-5-yl)-*N*^1^,*N*^4^-dipropylterephthalamide (**R3**) was obtained as a yellow solid. Yield 33%. ^1^H NMR (300 MHz, CDCl_3_) *δ* 7.31 (broad s Hz, 4H), 5.65 (s, 2H), 4.19 (broad s, 2H), 3.50–2.65 (m, 14H), 2.58 (m, 2H), 1.80 (m, 4H), 1.65–1.00 (m, 16H), 0.65 (broad s, 6H); ^13^C NMR (75 MHz, CDCl_3_) *δ* 173.8, 172.7, 171.4, 138.2, 126.4, 110.1, 90.8, 62.6, 59.9, 57.8, 53.6, 48.3, 33.6, 29.6, 27.2, 26.3, 24.1, 22.0, 11.1; MS (ESI, +ve) *m*/*z* 683 [(M + H)^+^, 100%]; HRMS (ESI, +ve) *m*/*z* [M + H]^+^ calcd for C_40_H_51_N_4_O_6_ 683.3803, found 683.3800.

##### *N*-((5*S*,6*S*,11a*S*,11b*S*)-2-Oxo-2,4,5,6,9,10,11,11a-octahydro-8*H*-6,11b-methanofuro[2,3-*c*]pyrido[1,2-*a*]azepin-5-yl)-2-(4-(2-oxo-2-(((5*S*,6*S*,11a*R*,11b*S*)-2-oxo-2,4,5,6,9,10,11,11a-octahydro-8*H*-6,11b-methanofuro[2,3-*c*]pyrido[1,2-*a*]azepin-5-yl)(propyl)amino)ethyl)phenyl)-*N*-propylacetamide (**R4**)

*N*-((5*S*,6*S*,11a*S*,11b*S*)-2-Oxo-2,4,5,6,9,10,11,11a-octahydro-8*H*-6,11b-methanofuro[2,3-*c*]pyrido[1,2-*a*]azepin-5-yl)-2-(4-(2-oxo-2-(((5*S*,6*S*,11a*R*,11b*S*)-2-oxo-2,4,5,6,9,10,11,11a-octahydro-8*H*-6,11b-methanofuro[2,3-*c*]pyrido[1,2-*a*]azepin-5-yl)(propyl)amino)ethyl)phenyl)-*N*-propylacetamide (**R4**) was obtained as a white solid. Yield 36%. ^1^H NMR (300 MHz, CDCl_3_) *δ* 7.23 (m, 1H), 7.05 (m, 3H), 5.64 (s, 2H), 4.28 (broadened s, 2H), 3.79–3.39 (m, 6H), 3.30–3.10 (m, 4H), 3.05–2.76 (m, 10H), 2.48 (broadened s, 2H), 1.92–1.15 (m, 18H), 0.87 (broadened s, 6H); ^13^C NMR (75 MHz, CDCl_3_) *δ* (mixture of rotamers) 174.4, 172.8, 171.3, 135.5, 129.3, 129.0, 127.4, 110.0, 91.1, 61.6, 60.0, 57.4, 48.4, 41.1, 33.4, 26.8, 26.4, 25.3, 24.1, 22.1, 11.3; MS (ESI, +ve) *m*/*z* 711 [(M + H)^+^, 97.2%]; HRMS (ESI, +ve) *m*/*z* [M + H]^+^ calcd for C_42_H_55_N_4_O_6_ 711.4116, found 711.4126.

##### 2,2′-(1,3-phenylene)bis(*N*-((5*S*,6*S*,11a*S*,11b*S*)-2-Oxo-2,4,5,6,9,10,11,11a-octahydro-8*H*-6,11b-methanofuro[2,3-*c*]pyrido[1,2-*a*]azepin-5-yl)-*N*-propylacetamide) (**R5**)

2,2′-(1,3-phenylene)bis(*N*-((5*S*,6*S*,11a*S*,11b*S*)-2-Oxo-2,4,5,6,9,10,11,11a-octahydro-8*H*-6,11b-methanofuro[2,3-*c*]pyrido[1,2-*a*]azepin-5-yl)-*N*-propylacetamide) (**R5**) was obtained as a yellow solid. Yield 32%. ^1^H NMR (300 MHz, CDCl_3_) *δ* 7.17 (s, 4H), 5.65 (s, 2H), 4.34 (m, 2H), 3.75–3.41 (m, 6H), 3.33–3.09 (m, 4H), 3.02–2.77 (m, 10H), 2.53 (m, 2H), 1.76–1.22 (m, 18H), 0.88 (m, 6H); ^13^C NMR (75 MHz, CDCl_3_) *δ* 174.4, 172.8, 171.5, 133.7, 129.1, 110.1, 91.1, 61.6, 60.0, 57.3, 53.5, 48.4, 40.8, 33.4, 26.8, 26.3, 25.4, 24.2, 22.1, 11.2 (signals due to two carbons obscured or overlapping); MS (ESI, +ve) *m*/*z* 711 [(M + H)^+^, 100%]; HRMS (ESI, +ve) *m*/*z* [M + H]^+^ calcd for C_42_H_55_N_4_O_6_ 711.4116, found 711.4126.

##### *N*-((5*S*,6*S*,11a*S*,11b*S*)-2-Oxo-2,4,5,6,9,10,11,11a-octahydro-8*H*-6,11b-methanofuro[2,3-*c*]pyrido[1,2-*a*]azepin-5-yl)-2-(2-(2-oxo-2-(((5*S*,6*S*,11a*R*,11b*S*)-2-oxo-2,4,5,6,9,10,11,11a-octahydro-8*H*-6,11b-methanofuro[2,3-*c*]pyrido[1,2-*a*]azepin-5-yl)(propyl)amino)ethyl)phenyl)-*N*-propylacetamide (**R6**)

*N*-((5*S*,6*S*,11a*S*,11b*S*)-2-Oxo-2,4,5,6,9,10,11,11a-octahydro-8*H*-6,11b-methanofuro[2,3-*c*]pyrido[1,2-*a*]azepin-5-yl)-2-(2-(2-oxo-2-(((5*S*,6*S*,11a*R*,11b*S*)-2-oxo-2,4,5,6,9,10,11,11a-octahydro-8*H*-6,11b-methanofuro[2,3-*c*]pyrido[1,2-*a*]azepin-5-yl)(propyl)amino)ethyl)phenyl)-*N*-propylacetamide (**R6**) was obtained as a white solid. Yield 43%. ^1^H NMR (300 MHz, CDCl_3_) *δ* 7.24–7.07 (m, 4H), 5.71 (m, 2H), 4.38 (m, 2H), 3.75–2.70 (m, 20H), 2.56 (m, 2H), 1.89–1.26 (m, 18H), 0.90 (m, 6H); ^13^C NMR (75 MHz, CDCl_3_) *δ* 174.1, 172.9, 171.4, 134.5, 129.5, 127.5, 110.2, 91.0, 61.5, 60.1, 57.2, 53.5, 48.4, 38.9, 33.4, 26.7, 26.4, 25.3, 24.1, 22.1, 11.2; MS (ESI, +ve) *m*/*z* 711 [(M + H)^+^, 100%]; HRMS (ESI, +ve) *m*/*z* [M + H]^+^ calcd for C_42_H_55_N_4_O_6_ 711.4116, found 711.4126.

##### (1*R*,2*S*)-*N*^1^-((5*S*,6*S*,11a*R*,11b*S*)-2-Oxo-2,4,5,6,9,10,11,11a-octahydro-8*H*-6,11b-methanofuro[2,3-*c*]pyrido[1,2-*a*]azepin-5-yl)-*N*^2^-((5*S*,6*S*,11a*S*,11b*S*)-2-oxo-2,4,5,6,9,10,11,11a-octahydro-8*H*-6,11b-methanofuro[2,3-*c*]pyrido[1,2-*a*]azepin-5-yl)-*N*^1^,*N*^2^-dipropylcyclohexane-1,2-dicarboxamide(**HR1**)

(1*R*,2*S*)-*N*^1^-((5*S*,6*S*,11a*R*,11b*S*)-2-Oxo-2,4,5,6,9,10,11,11a-octahydro-8*H*-6,11b-methanofuro[2,3-*c*]pyrido[1,2-*a*]azepin-5-yl)-*N*^2^-((5*S*,6*S*,11a*S*,11b*S*)-2-oxo-2,4,5,6,9,10,11,11a-octahydro-8*H*-6,11b-methanofuro[2,3-*c*]pyrido[1,2-*a*]azepin-5-yl)-*N*^1^,*N*^2^-dipropylcyclohexane-1,2-dicarboxamide(**HR1**) was obtained as a white solid. Yield 21%. ^1^H NMR (300 MHz, CDCl_3_) *δ* 5.56 (m, 2H), 4.30 (m, 2H), 3.75–2.35 (m, 18H), 2.30–1.00 (m, 28H), 0.89–0.80 (m, 6H); ^13^C NMR (75 MHz, CDCl_3_) *δ* (mixture of rotamers) 175.3, 175.2, 174.6, 173.2, 172.8, 110.2, 109.9, 91.4, 91.1, 60.2, 59.9, 56.6, 48.5, 48.3, 41.9, 33.3, 30.1, 29.1, 27.0, 26.6, 26.5, 26.1, 24.3, 24.2, 23.3, 23.1, 22.2, 14.1, 11.3, 11.2, 11.1; MS (ESI, +ve) *m*/*z* 689 [(M + H)^+^, 100%]; HRMS (ESI, +ve) *m*/*z* [M + H]^+^ calcd for C_40_H_57_N_4_O_6_ 689.4273, found 689.4274.

##### (1*R*,3*S*)-*N*^1^-((5*S*,6*S*,11a*R*,11b*S*)-2-Oxo-2,4,5,6,9,10,11,11a-octahydro-8*H*-6,11b-methanofuro[2,3-*c*]pyrido[1,2-*a*]azepin-5-yl)-*N*^3^-((5*S*,6*S*,11a*S*,11b*S*)-2-oxo-2,4,5,6,9,10,11,11a-octahydro-8*H*-6,11b-methanofuro[2,3-*c*]pyrido[1,2-*a*]azepin-5-yl)-*N*^1^,*N*^3^-dipropylcyclohexane-1,3-dicarboxamide(**HR2**)

(1*R*,3*S*)-*N*^1^-((5*S*,6*S*,11a*R*,11b*S*)-2-Oxo-2,4,5,6,9,10,11,11a-octahydro-8*H*-6,11b-methanofuro[2,3-*c*]pyrido[1,2-*a*]azepin-5-yl)-*N*^3^-((5*S*,6*S*,11a*S*,11b*S*)-2-oxo-2,4,5,6,9,10,11,11a-octahydro-8*H*-6,11b-methanofuro[2,3-*c*]pyrido[1,2-*a*]azepin-5-yl)-*N*^1^,*N*^3^-dipropylcyclohexane-1,3-dicarboxamide(**HR2**) was obtained as a yellow oil. Yield 26%. ^1^H NMR (300 MHz, CDCl_3_) *δ* 5.65 (broad s, 2H), 4.33 (m, 2H), 3.41–2.60 (m, 14H), 2.54–2.23 (m, 4H), 1.95–1.10 (m, 28H), 0.92–0.73 (m, 6H); ^13^C NMR (75 MHz, CDCl_3_) *δ* (mixture of rotamers) 175.6, 175.5, 174.5, 174.4, 172.7, 172.7, 110.1, 93.5, 91.1, 91.0, 61.5, 61.4, 59.9(4), 59.8(9), 56.7, 56.6, 53.5, 48.3(7), 48.3(5), 48.3(3), 47.5(7), 47.5(6), 41.2, 40.7, 33.2, 29.6, 26.4, 26.2(4), 26.2(1), 22.1(7), 22.1(0), 11.2(4), 11.1(5); MS (ESI, +ve) *m*/*z* 689 [(M + H)^+^, 100%]; HRMS (ESI, +ve) *m*/*z* [M + H]^+^ calcd for C_40_H_57_N_4_O_6_ 689.4273, found 689.4274.

##### (1*S*,4*S*)-*N*^1^,*N*^4^-bis((5*S*,6*S*,11a*S*,11b*S*)-2-Oxo-2,4,5,6,9,10,11,11a-octahydro-8*H*-6,11b-methanofuro[2,3-*c*]pyrido[1,2-*a*]azepin-5-yl)-*N*^1^,*N*^4^-dipropylcyclohexane-1,4-dicarboxamide (**HR3**)

(1*S*,4*S*)-*N*^1^,*N*^4^-bis((5*S*,6*S*,11a*S*,11b*S*)-2-Oxo-2,4,5,6,9,10,11,11a-octahydro-8*H*-6,11b-methanofuro[2,3-*c*]pyrido[1,2-*a*]azepin-5-yl)-*N*^1^,*N*^4^-dipropylcyclohexane-1,4-dicarboxamide (**HR3**) was obtained as a white solid. Yield 8%. ^1^H NMR (300 MHz, CDCl_3_) *δ* 5.65 (m, 2H), 4.28–4.05 (m, 2H), 3.35–2.25 (m, 20H), 1.90–1.05 (m, 26H), 0.76 (m, 6H); ^13^C NMR (75 MHz, CDCl_3_) *δ* (mixture of rotamers) 176.2, 174.4, 172.7, 110.0, 91.0, 61.7, 60.0, 57.1, 53.5, 48.3, 48.0, 40.6, 33.2, 29.4, 28.1, 26.8, 26.4, 25.9, 24.1, 22.1, 11.1; MS (ESI, +ve) *m*/*z* 689 [(M + H)^+^, 100%]; HRMS (ESI, +ve) *m*/*z* [M + H]^+^ calcd for C_40_H_57_N_4_O_6_ 689.4273, found 689.4274.

### 4.2 Modeling and biological evaluation

#### 4.2.1 Docking studies

The Topo I structure incorporated within the SYBYL software package (*ex*. Tripos Inc. St. Louis, MO, USA) was used and all the associated water and small molecule ligands were removed. Modifications necessary to simulate a physiological environment^22^ were also made. Docking processes were refined using the SYBYL-8.1 surflex-docking module and a ligand-based approach was used to generate the binding site of the receptor. The protocol was generated with a threshold parameter of 0.5 Å and a bloat parameter of 0 Å while all Kollman charges were appended and the GeomX mode was applied to obtain better outcomes in the docking study. Potential ligands were docked sequentially into the binding pocket. Default settings were used for all other relevant parameters.

#### 4.2.2 Topo I inhibitory assay

In this assay, CPT was used as the positive control^23^ and the degree of inhibition of calf thymus Topo I (*ex*. TaKaRa, Kyoto, Japan) reflected in the relative amount of supercoiled pBR322 DNA (TaKaRa, Kyoto, Japan) remaining at the endpoint.^24^ Relevant solutions were prepared as defined in the supplier's protocols. Incubations were carried out at 37 °C for 0.5 h then a staining solution, comprising 0.25% w/v bromophenol blue, 0.25% w/v xylene cyanol FF and 40% v/v glycerol in doubly distilled water, was added and so terminating the reaction. The final mixtures were submitted to electrophoresis for 0.66 h in 1× TAE buffer (comprising 40 mM tris-acetate, 2 mM EDTA and 19.9 mM HOAc) in 1% agarose gel. DNA bands were visualized through illumination with UV light and photographed with an Alpha Innotech digital imaging system after staining with 5 μg mL^–1^ EB in 1× TAE buffer for 0.5 h.

#### 4.2.3 Electrophoretic mobility shift assays (EMSAs)

In a representative experiment,^15^ 1 μL BSA was added into 20 μL of the Topo I buffer. Four units of calf thymus Topo I and compound **R2** were then added and the resulting mixture allowed to stand for 0.33 h. Finally, 0.1 μg of pBR322 DNA was added to the mixture that was then immersed in a water bath at 37 °C for 0.5 h. After this time the mixture was immediately subjected to electrophoresis at 1 V cm^–1^ and 4 °C for 8 h on 1% gel containing 1% EB. The DNA bands were visualized as described at 4.2.2.

#### 4.2.4 DNA-cleavage assay

20 units of Topo I, 0.5 μg of supercoiled pBR322 DNA, and 50 or 100 μM solutions of CPT or the test compounds were combined to a total volume of 40 μL in Topo I buffer (as provided by the supplier). Cleavage intermediates were trapped by adding 1% sodium dodecyl sulfate (SDS) to terminate the reaction after incubation at 37 °C for 0.5 h. Then 0.8 mg mL^–1^ proteinase K (pH 8.0) was added to the reaction mixture that was then incubated at 50 °C for 10 min (so as to digest the Topo I). 10 μl of samples of the resulting solution were mixed with 2 μL 6× DNA loading buffer, comprising 0.25% bromophenol blue, 0.25% xylene cyanol FF and 40% v/v glycerol in doubly distilled water, then submitted to electrophoresis at 60 V and 25 °C for 0.5 h. The gel was soaked in 1× TAE containing 0.5 μg mL^–1^ EB for 0.5 h then a second electrophoretic procedure of 0.5 h duration was conducted so as to distinguish the relaxed from the nicked forms of DNA. The cleavage effects were evaluated by comparing the relative intensities of nicked and other DNA bands that had been visualized by illumination with UV light then photographed as described at 4.2.2.

#### 4.2.5 DNA-insertion assay

This assay resembled the Topo I inhibitory one but varied in the amount of enzyme added. EB, a DNA intercalating agent,^25^ was used as the positive control. Thus, ten units of calf thymus Topo I (*ex*. TaKaRa, Kyoto, Japan), 0.1 μg of negatively supercoiled pBR322 DNA (TaKaRa, Kyoto, Japan) and 100 μM of the **R** series compound (or 25 μM of EB) were added into a total of 20 μL of Topo I buffer (as provided by the supplier). The ensuing mixture was combined with 4 μL of 6× DNA loading buffer after incubation at 37 °C for 0.5 h. Samples were subjected to electrophoresis for 0.66 h in the same manner as defined at 4.2.2 and DNA bands were visualized and recorded in the usual way.

## Supplementary Material

Supplementary information

## References

[cit1] Wang J. C. (1971). J. Mol. Biol..

[cit2] Ross W. E. (1985). Biochem. Pharmacol..

[cit3] Hsiang Y.-H., Hertzberg R., Hecht S., Liu L. F. (1985). J. Biol. Chem..

[cit4] Roberts J. K., Birg A. V., Lin T., Daryani V. M., Panetta J. C., Broniscer A., Robinson G. W., Gajjar A. J., Stewart C. F. (2016). Drug Metab. Dispos..

[cit5] Hu Q., Wang Q., Zhu H., Yao Y., Song Q. (2016). J. Cancer Res. Ther..

[cit6] Hou W., Wang Z.-Y., Peng C.-K., Lin J., Liu X., Chang Y.-Q., Xu J., Jiang R.-W., Lin H., Sun P.-H., Chen W.-M. (2016). Eur. J. Med. Chem..

[cit7] Gaur R., Pathania A. S., Malik F. A., Bhakuni R. S., Verma R. K. (2016). Eur. J. Med. Chem..

[cit8] Dunlap N., Salyard T. L. J., Pathiranage A. L., Stubblefield J., Pitts S. L., Ashley R. E., Osheroff N. (2014). Bioorg. Med. Chem. Lett..

[cit9] Venkatraj M., Ariën K. K., Heeres J., Joossens J., Messagie J., Michael J., Veken P. V. D., Vanham G., Lewi P. J., Augustyns K. (2012). Bioorg. Med. Chem. Lett..

[cit10] Dunlap N., Salyard T. L. J., Pathiranage A. L., Stubblefield J., Pitts S. L., Ashley R. E., Osheroff N. (2014). Bioorg. Med. Chem. Lett..

[cit11] Susova O. Y., Ivanov A. A., Ruiz S. S. M., Lesovaya E. A., Gromyko A. V., Streltsov S. A., Zhuze A. L. (2010). Biochemistry.

[cit12] Wang Z. H., Wu Z. J., Yue D. F., You Y., Xu X. Y., Zhang X. M., Yuan W. C. (2016). Org. Biomol. Chem..

[cit13] D'yakonov V. A., Dzhemileva L. U., Makarov A. A., Mulukova A. R., Baev D. S., Khusnutdinova E. K., Tolstikova T. G., Dzhemilev U. M. (2015). Bioorg. Med. Chem. Lett..

[cit14] Vadwai V., Devaraj S. (2009). Int. J. Bioinf. Res. Appl..

[cit15] Wu N., Wu X.-W., Agama K., Pommier Y., Du J., Li D., Gu L.-Q., Huang Z.-S., An L.-K. (2010). Biochemistry.

[cit16] Trask D. K., DiDonato J. A., Muller M. T. (1984). EMBO J..

[cit17] Gromova I. L., Kjeldsen E., Svejstrup J. Q., Alsner J., Christiansen K., Westergaard O. (1993). Nucleic Acids Res..

[cit18] Champoux J. J. (2001). Annu. Rev. Biochem..

[cit19] Singh A., Nepali K., Kaur N., Singh G., Sharma S., Sharma P. (2016). Recent Pat. Anticancer Drug Discov..

[cit20] Still W. C., Kahn M., Mitra A. (1978). J. Org. Chem..

[cit21] Tang G., Liu X., Ma N., Huang X., Wu Z.-L., Zhang W., Wang Y., Zhao B.-X., Wang Z.-Y., Ye W.-C., Shi L., Chen W.-M. (2016). ACS Chem. Neurosci..

[cit22] Kumar A., Bora U. (2014). Interdiscip. Sci.: Comput. Life Sci..

[cit23] Wang M.-J., Liu Y.-Q., Chang L.-C., Wang C.-Y., Zhao Y.-L., Zhao X.-B., Qian K., Nan X., Yang L., Yang X.-M., Hung H.-Y., Yang J.-S., Kuo D.-H., Goto M., Morris-Natschke S. L., Pan S.-L., Teng C.-M., Kuo S.-C., Wu T.-S., Wu Y.-C., Lee K.-H. (2014). J. Med. Chem..

[cit24] Yao B.-L., Mai Y., Chen S.-B., Xie H., Yao P.-F., Ou T., Tan J., Wang H., Li D., Huang S.-L., Gu L.-Q., Huang Z.-S. (2015). Eur. J. Med. Chem..

[cit25] Pulleyblank D. E., Morgan A. R. (1975). J. Mol. Biol..

