# Supplementary material for: Novel bivalent securinine mimetics as topoisomerase I inhibitors
Source: Medchemcomm. 2017 Jan 3;8(2):320–8. doi: 10.1039/c6md00563b (PMC6072210; doi:10.1039/c6md00563b)
Supplement: Supplementary file 1 [file MD-008-C6MD00563B-s001.pdf]

Electronic Supplementary Material (ESI) for MedChemComm.
This journal is © The Royal Society of Chemistry 2017

Supplementary Material

Novel bivalent securinine mimetics as topoisomerase I inhibitors

Wen Hou[1], Hui Lin[1], Zhen-Ya Wang[1], Martin G. Banwell[2], Ting Zeng[1], Ping-Hua Sun[1], Jing Lin[1,*] and Wei-Min Chen[1,*]

1. College of Pharmacy, Jinan University, Guangzhou 510632, P. R. China

2. Research School of Chemistry, Institute of Advanced Studies, The Australian National University, Canberra, ACT 2601, Australia

**Corresponding authors. Tel.: +86 20 8522 1367(J. Lin), +86 20 8522 4497 (W.-M. Chen).*

Fax: +86 20 8522 4766.

E-mail address: linjing_jnu@163.com (J. Lin), twmchen@jnu.edu.cn(W.-M. Chen).

Supplementary Material Contents:

^{1}H NMR-Spectra, ^{13}C NMR-spectra, LRMS or HRMS of target compounds

^{1}H NMR spectrum of **KA** (300 MHz, $CDCl_3$)

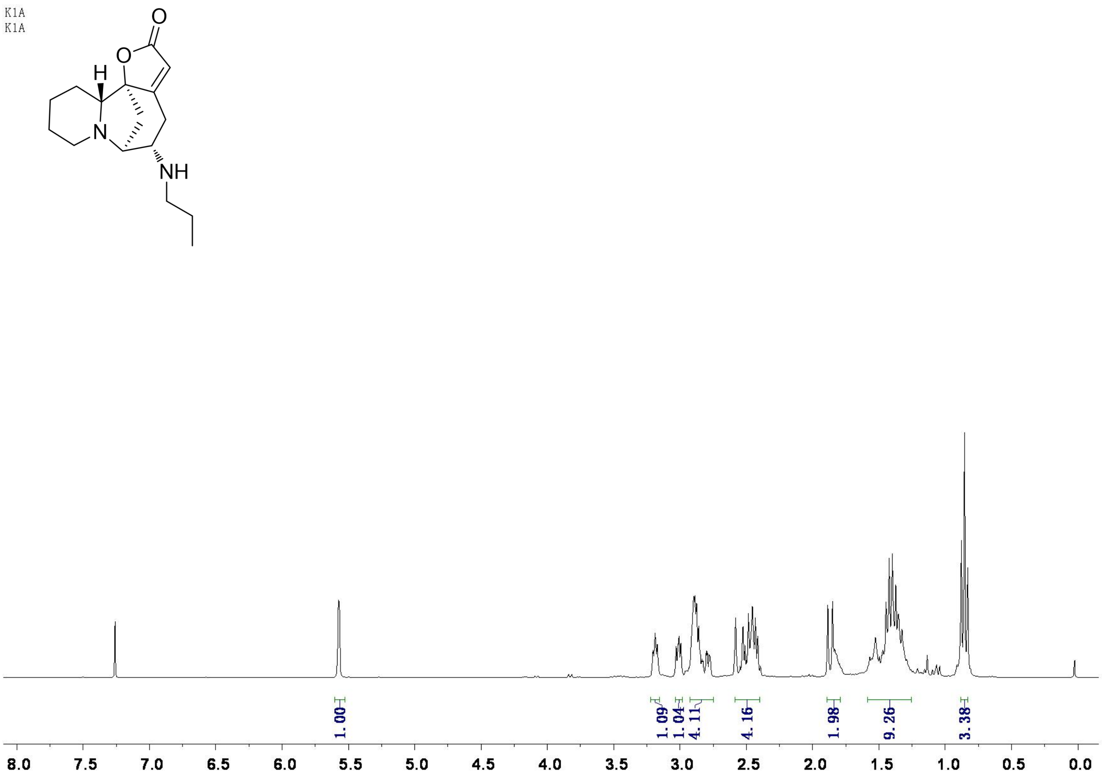

^{13}C NMR spectrum of **KA** (75 MHz, $CDCl_3$)

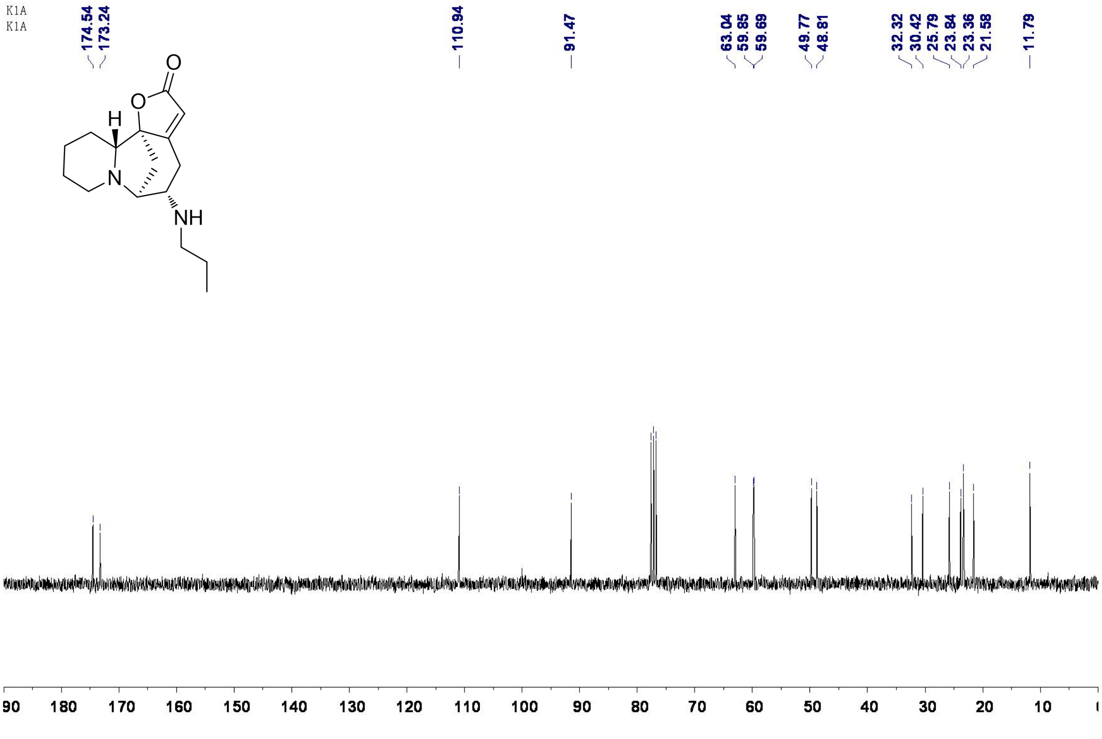

^{1}H NMR spectrum of **M2** (300 MHz, $CDCl_3$)

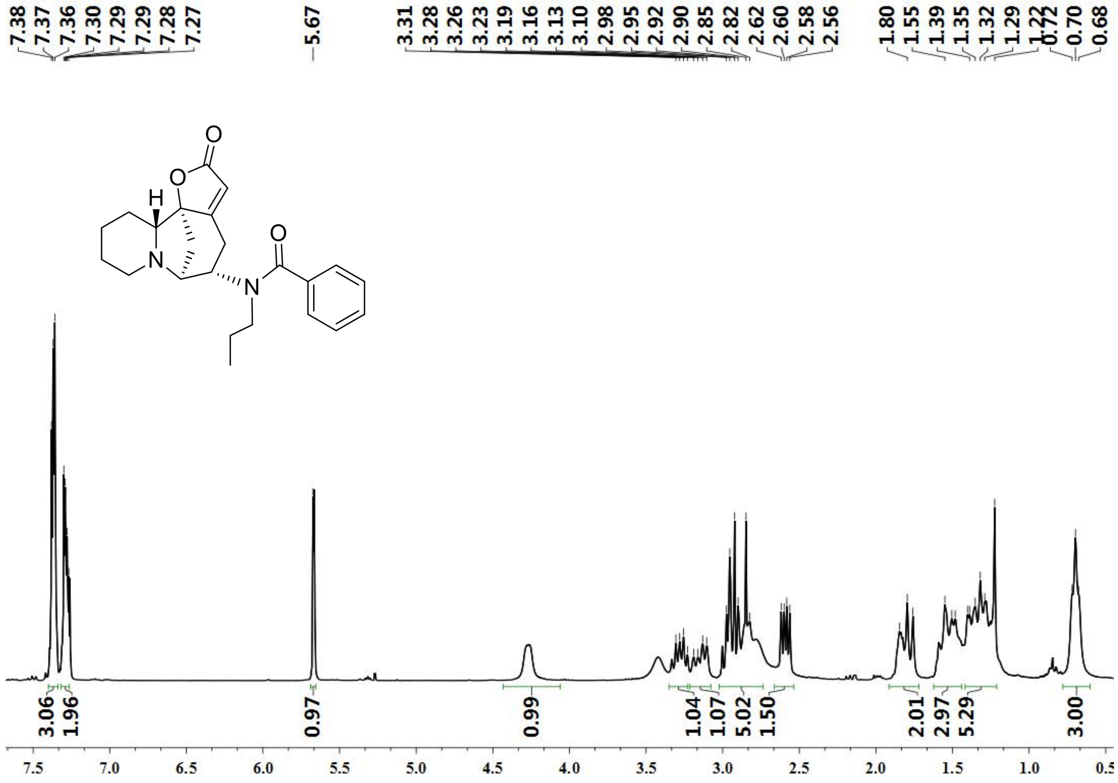

^{13}C NMR spectrum of **M2** (75 MHz, $CDCl_3$)

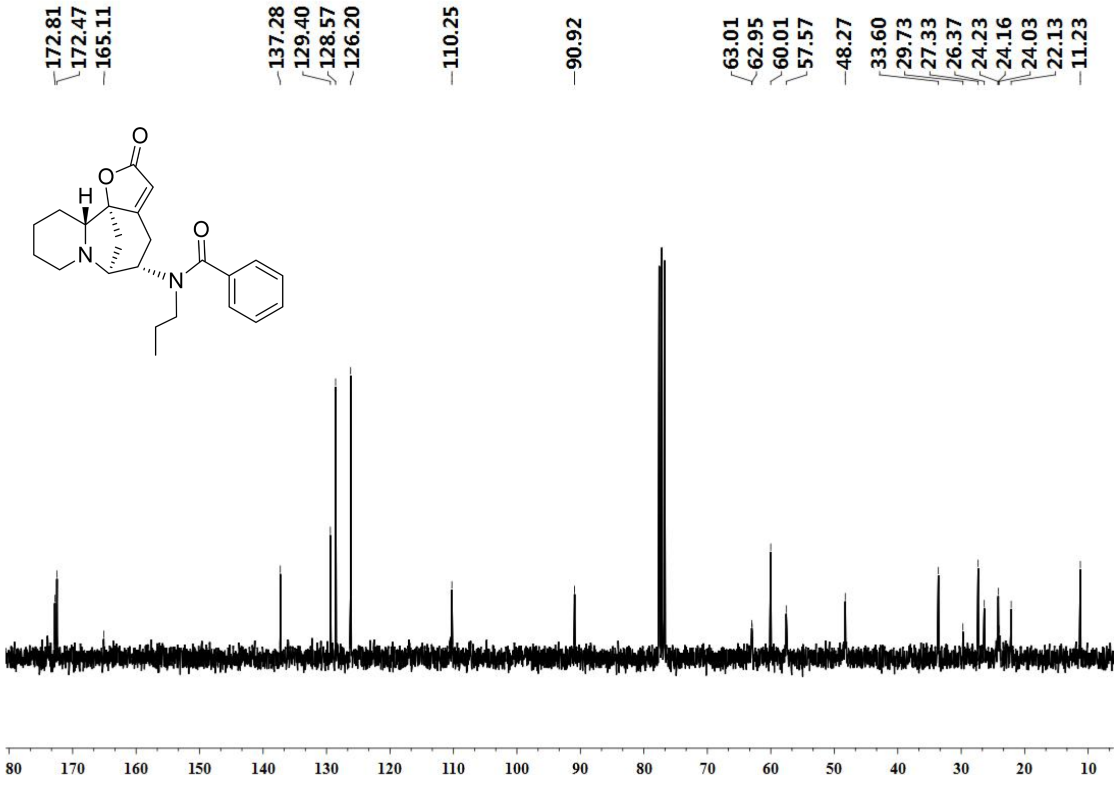

ESI-MS of compound **M2**

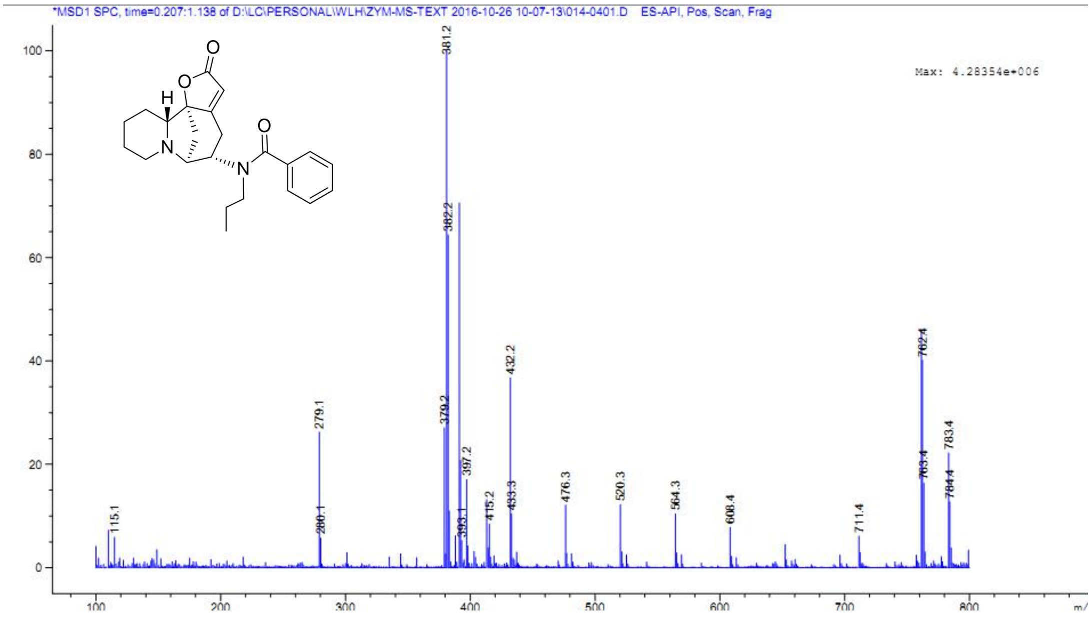

HRMS of compound **M2**

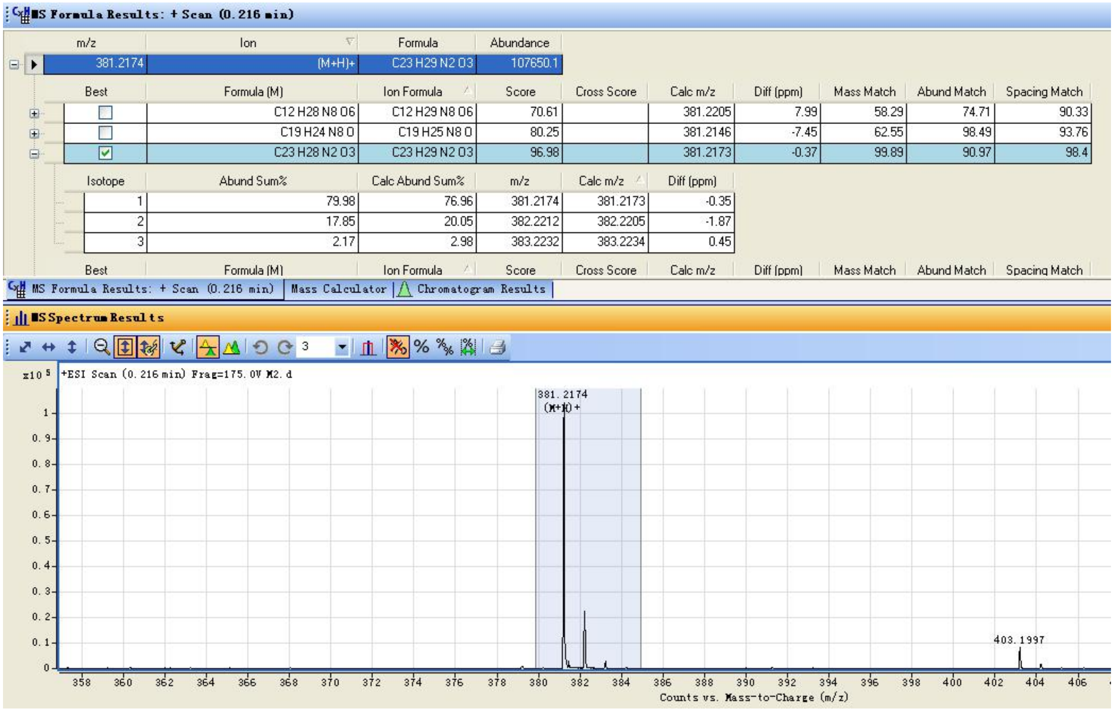

m/z	Ion	Formula	Abundance
381.2174	(M+H)+	C23 H29 N2 O3	107650.1

Best	Formula (M)	Ion Formula	Score	Cross Score	Calc m/z	Diff (ppm)	Mass Match	Abund Match	Spacing Match
☐	C12 H28 N8 O6	C12 H29 N8 O6	70.61		381.2205	7.99	58.29	74.71	90.33
☐	C19 H24 N8 O	C19 H25 N8 O	80.25		381.2146	-7.45	62.55	98.49	93.76
☑	C23 H28 N2 O3	C23 H29 N2 O3	96.98		381.2173	-0.37	99.89	90.97	98.4

Isotope	Abund Sum%	Calc Abund Sum%	m/z	Calc m/z	Diff (ppm)
1	79.98	76.96	381.2174	381.2173	-0.35
2	17.85	20.05	382.2212	382.2205	-1.87
3	2.17	2.98	383.2232	383.2234	0.45

^{1}H NMR spectrum of **R1** (300 MHz, $CDCl_3$)

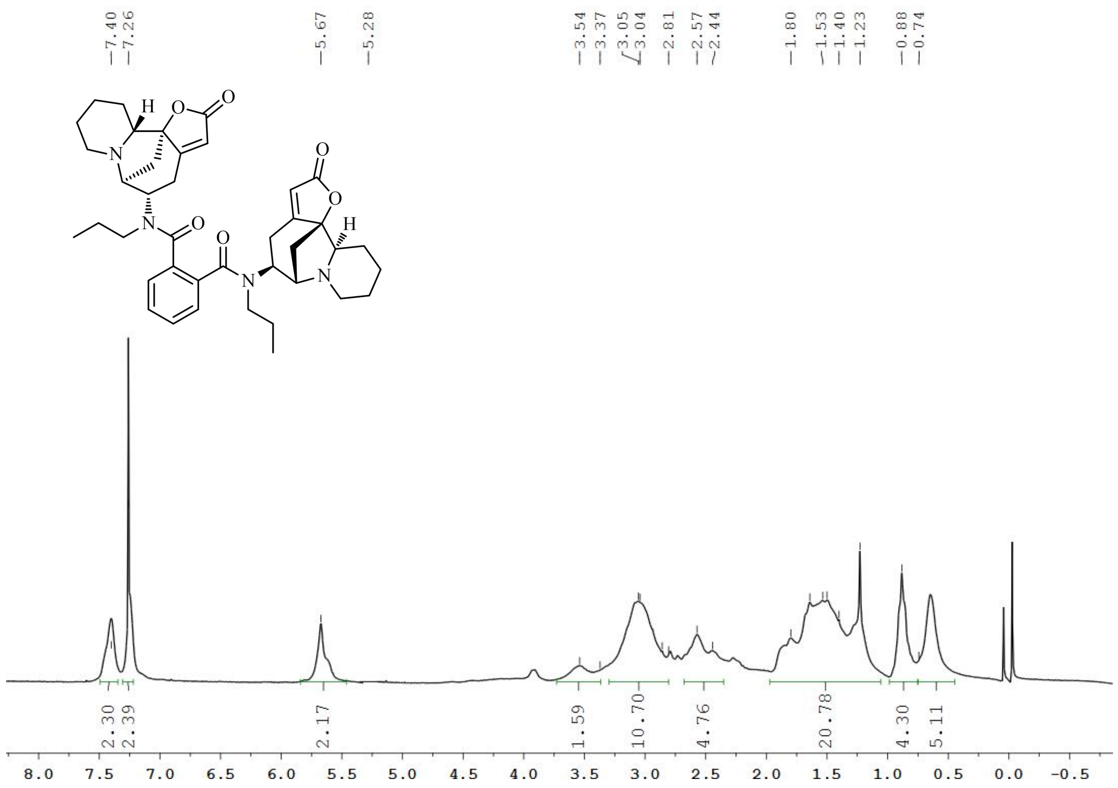

^{13}C NMR spectrum of **R1** (75 MHz, $CDCl_3$)

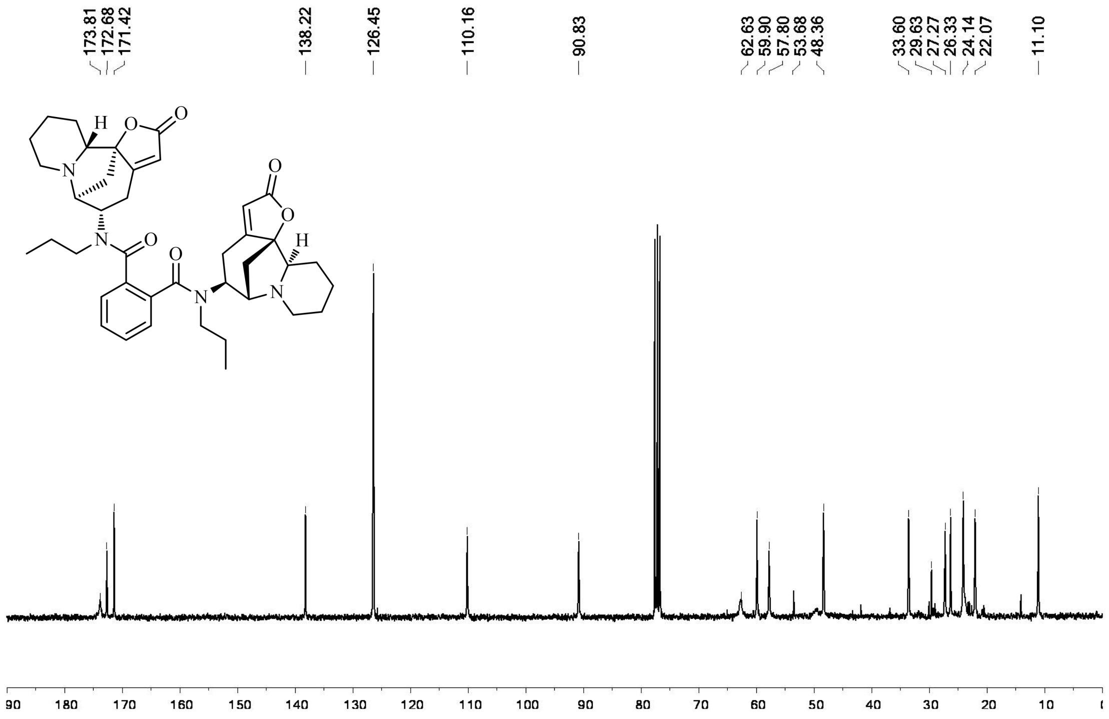

ESI-MS spectrum of **R1**

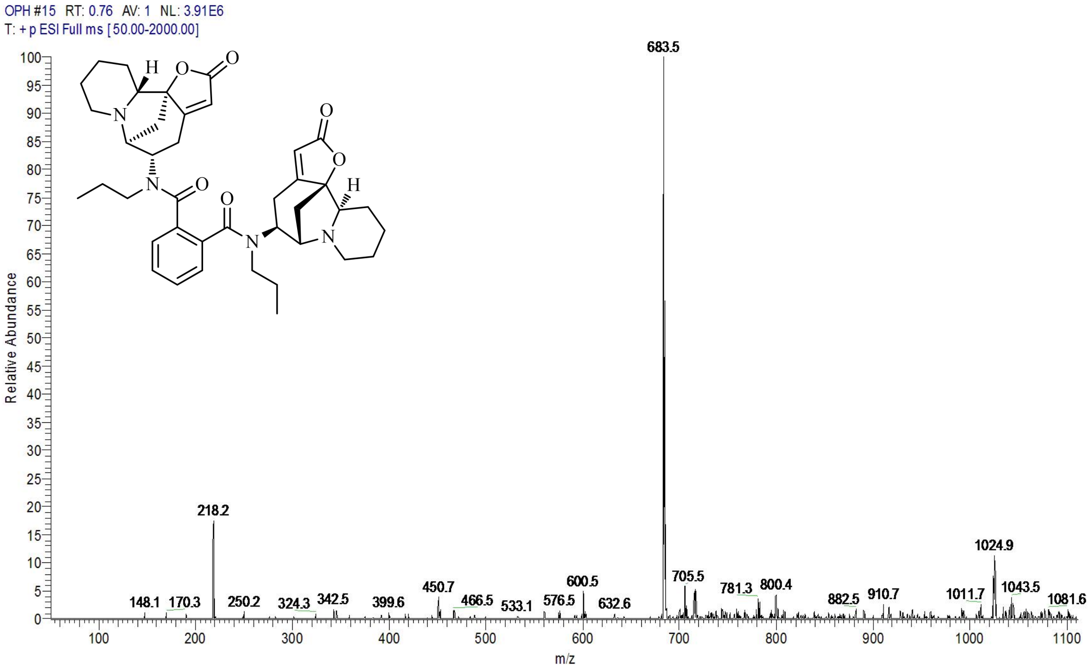

HRMS of compound **R1**

MS Formula Results: + Scan (0.253 min)

Best	Formula (M)	Ion Formula	Score	Cross Score	Calc m/z	Diff (ppm)	Mass Match	Abund Match	Spacing Match
☑	C40 H50 N4 O6	C40 H51 N4 O6	99.41		683.3803	-0.4	99.78	98.98	99.2

Isotope	Abund Sum%	Calc Abund Sum%	m/z	Calc m/z	Diff (ppm)
1	64.02	62.92	683.3806	683.3803	-0.38
2	28.01	28.65	684.3839	684.3835	-0.61
3	6.69	7.15	685.3859	685.3865	0.84
4	1.27	1.28	686.3871	686.3893	3.26

Best	Formula (M)	Ion Formula	Score	Cross Score	Calc m/z	Diff (ppm)	Mass Match	Abund Match	Spacing Match
☐	C41 H46 N8 O2	C41 H47 N8 O2	96.89		683.3816	1.54	96.8	95.04	99.3
☐	C45 H50 N2 O4	C45 H51 N2 O4	80.41		683.3843	5.51	65.96	89.3	98.63
☐	C46 H46 N6	C46 H47 N6	69.08		683.3857	7.45	46.73	81.42	98.97

MS Formula Results: + Scan (0.253 min) | Mass Calculator | Chromatogram Results

MS Spectrum Results

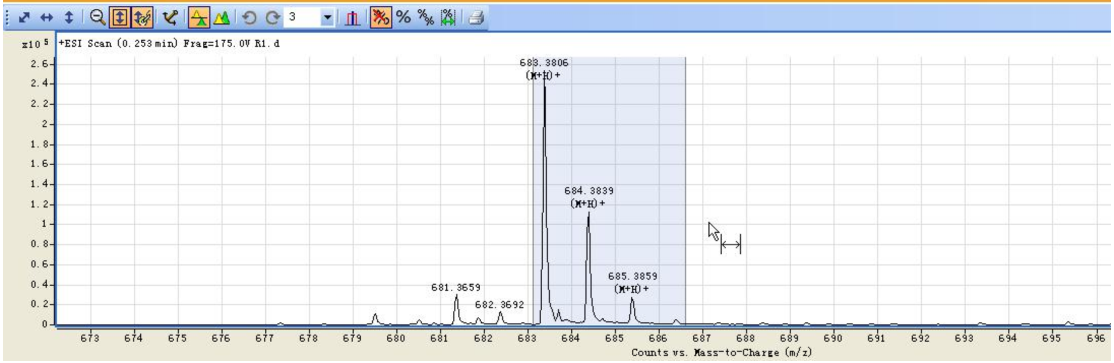

H NMR spectrum of **R2** (300 MHz, $CDCl_3$)

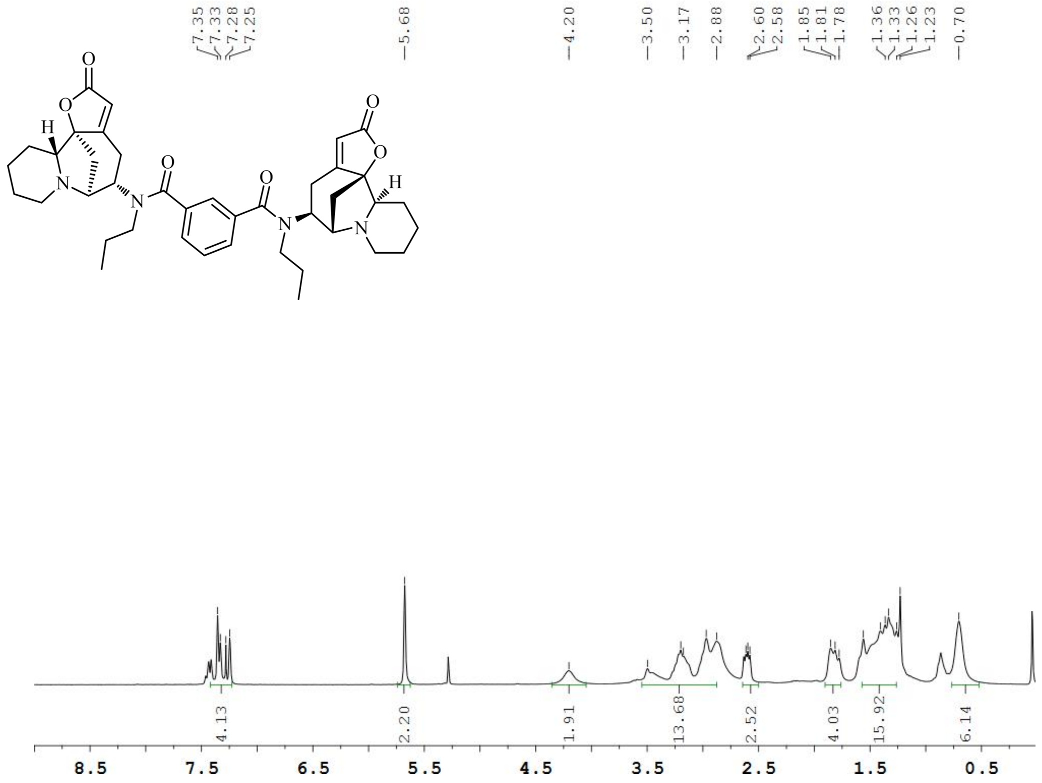

^{13}C NMR spectrum of **R2** (75 MHz, $CDCl_3$)

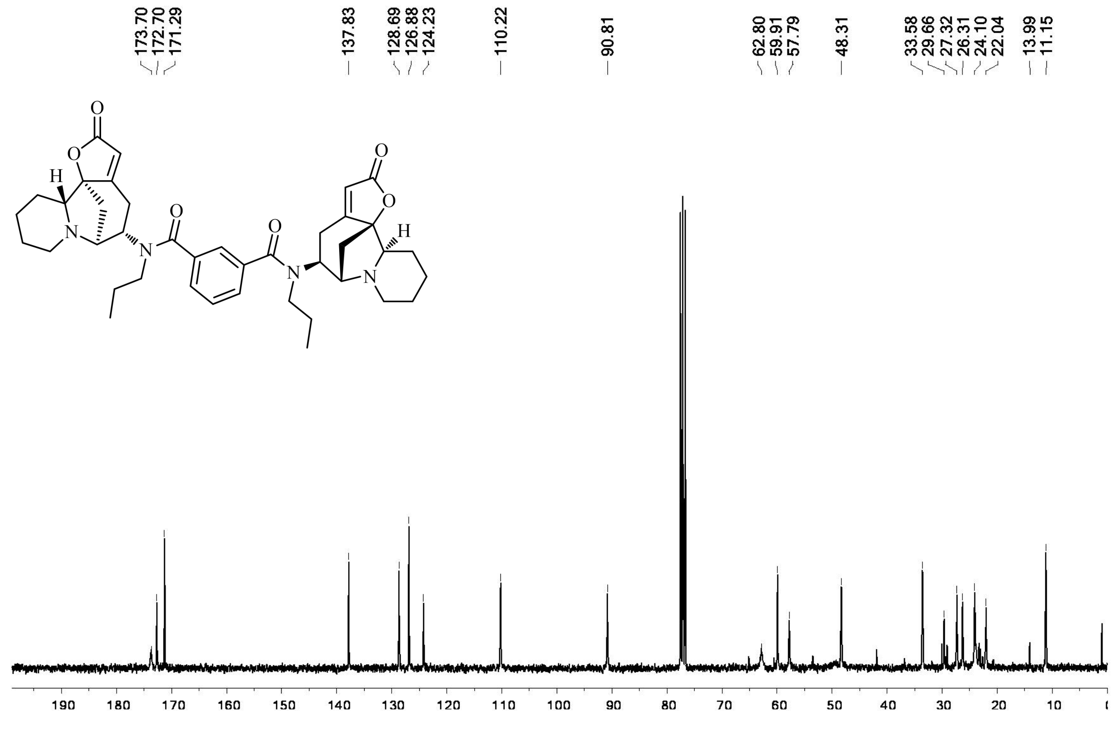

ESI-MS spectrum of **R2**

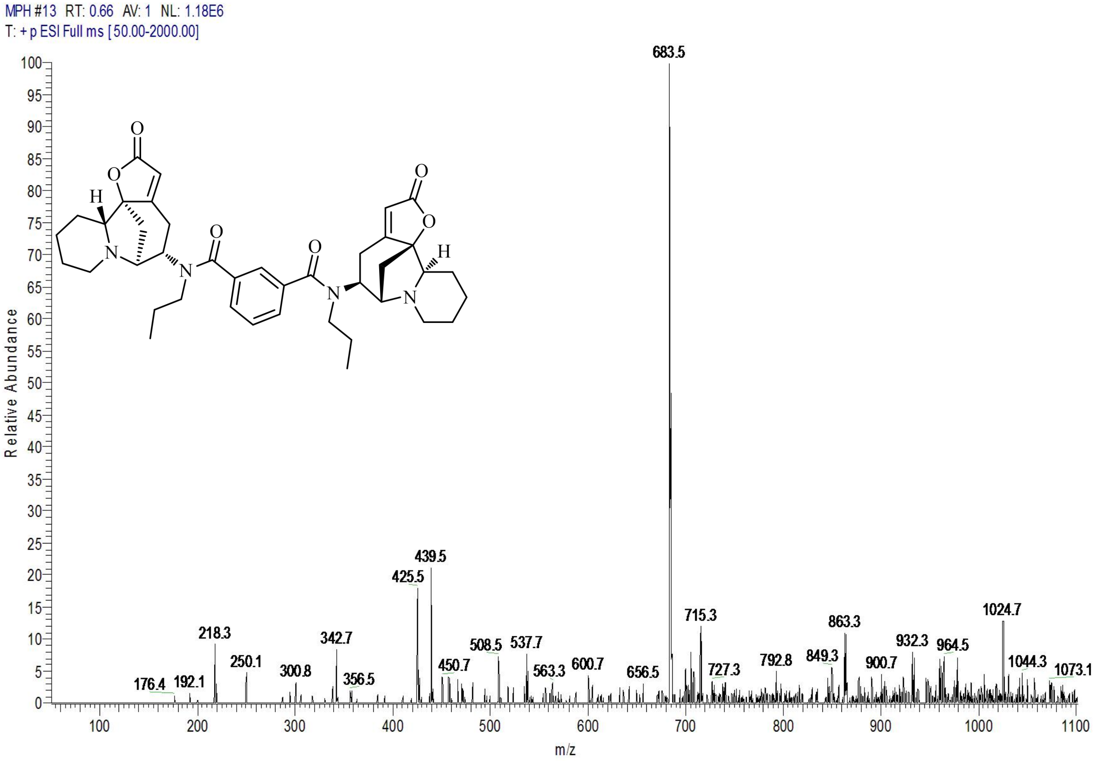

HRMS of compound **R2**

m/z	Ion	Formula	Abundance
683.381	(M+H)+	C40 H51 N4 O6	6293.6

Best	Formula (M)	Ion Formula	Calc m/z	Score	Mass	Calc Mass	Diff (ppm)	Abs Diff (ppm)	Abund Match	Spacing Match	Mass Match	m/z	DBE
☑	C40 H50 N4 O6	C40 H51 N4 O6	683.3803	87.34	682.3731	682.373	-0.12	0.12	93.93	54.17	99.98	683.381	18

Isotope	Abund%	Calc Abund%	Calc Abund Sum%	m/z	Calc m/z	Diff (ppm)	Abund Sum%
1	100	100	63.73	683.381	683.3803	-1.01	62.46
2	46.56	45.54	29.02	684.3775	684.3835	8.76	29.08
3	13.54	11.37	7.24	685.3902	685.3865	-5.38	8.46

Best	Formula (M)	Ion Formula	Calc m/z	Score	Mass	Calc Mass	Diff (ppm)	Abs Diff (ppm)	Abund Match	Spacing Match	Mass Match	m/z	DBE

Mass Calculator | Chromatogram Results | MS Formula Results: + Scan (0.397 min)

MS Spectrum Results

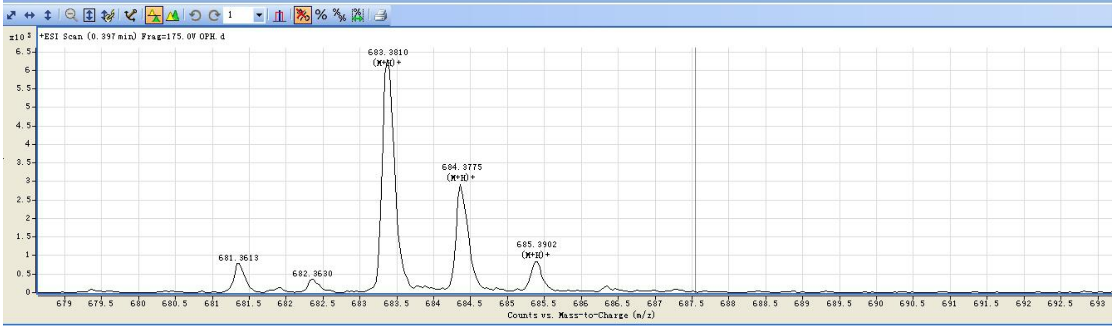

^{1}H NMR spectrum of **R3** (300 MHz, $CDCl_3$)

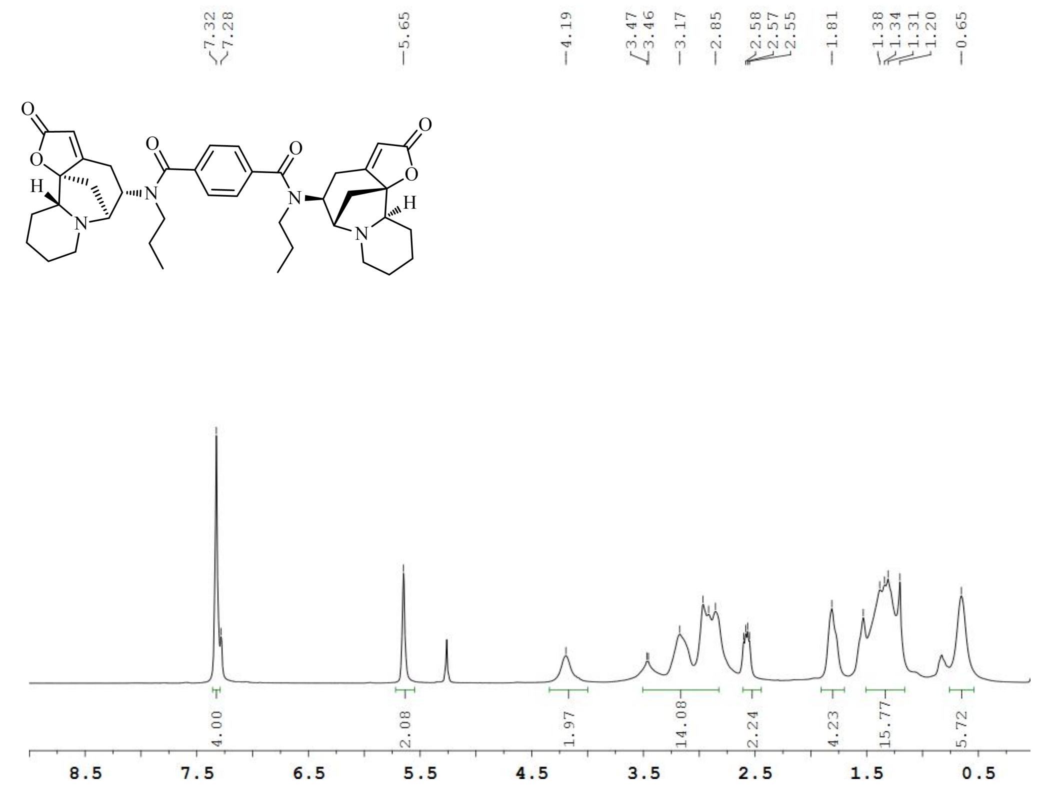

^{13}C NMR spectrum of **R3** (75 MHz, $CDCl_3$)

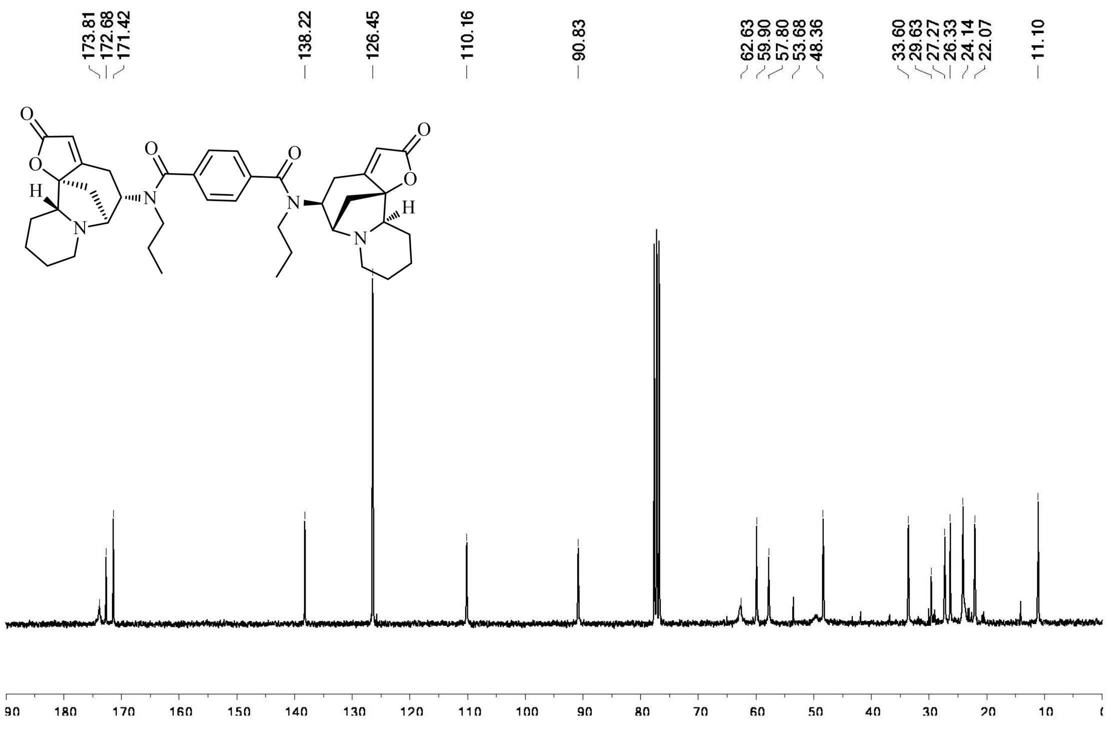

ESI-MS spectrum of **R3**

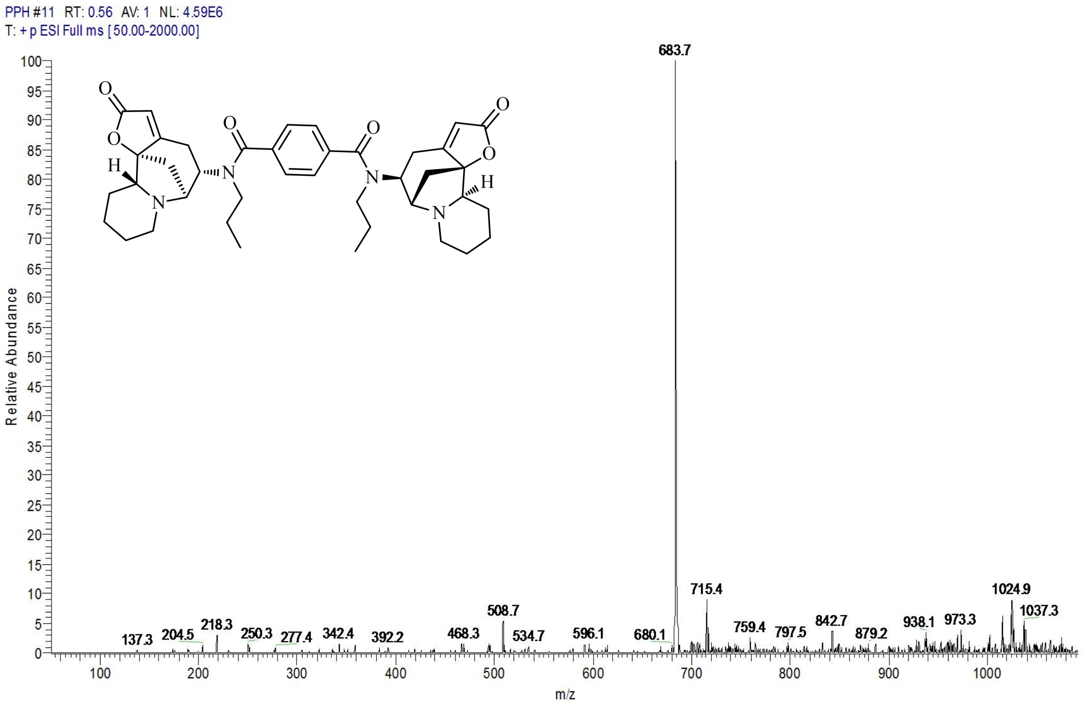

HRMS of compound **R3**

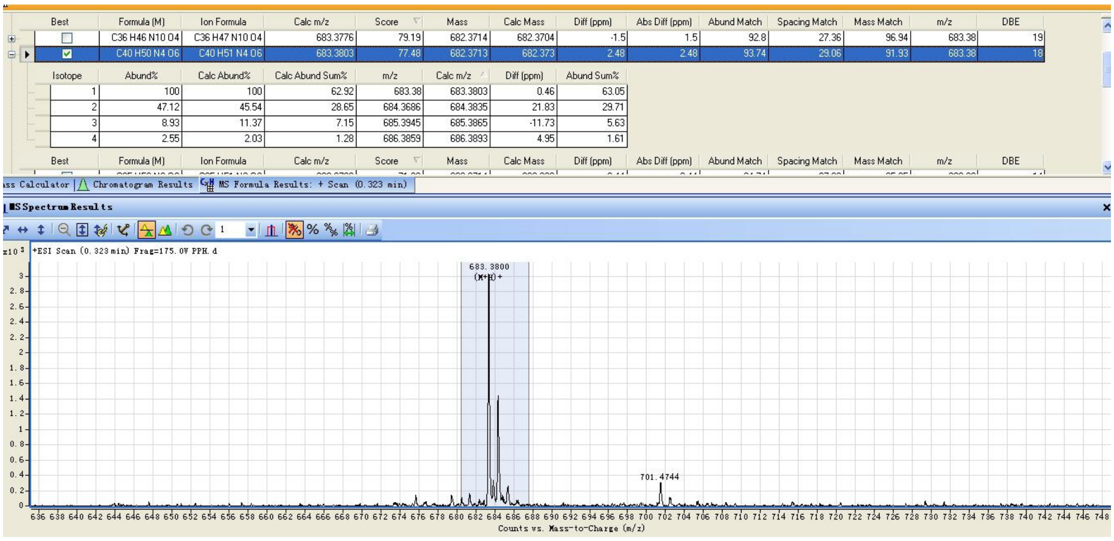

Best	Formula (M)	Ion Formula	Calc m/z	Score	Mass	Calc Mass	Diff (ppm)	Abs Diff (ppm)	Abund Match	Spacing Match	Mass Match	m/z	DBE
☐	C36 H46 N10 O4	C36 H47 N10 O4	683.3776	79.19	682.3714	682.3704	-1.5	1.5	92.8	27.36	96.94	683.38	19
☑	C40 H50 N4 O6	C40 H51 N4 O6	683.3803	77.48	682.3713	682.373	2.48	2.48	93.74	29.06	91.93	683.38	18

Isotope	Abund%	Calc Abund%	Calc Abund Sum%	m/z	Calc m/z	Diff (ppm)	Abund Sum%
1	100	100	62.92	683.38	683.3803	0.46	63.05
2	47.12	45.54	28.65	684.3686	684.3835	21.83	29.71
3	8.93	11.37	7.15	685.3945	685.3865	-11.73	5.63
4	2.55	2.03	1.28	686.3859	686.3893	4.95	1.61

^{1}H NMR spectrum of **R4** (300 MHz, $CDCl_3$)

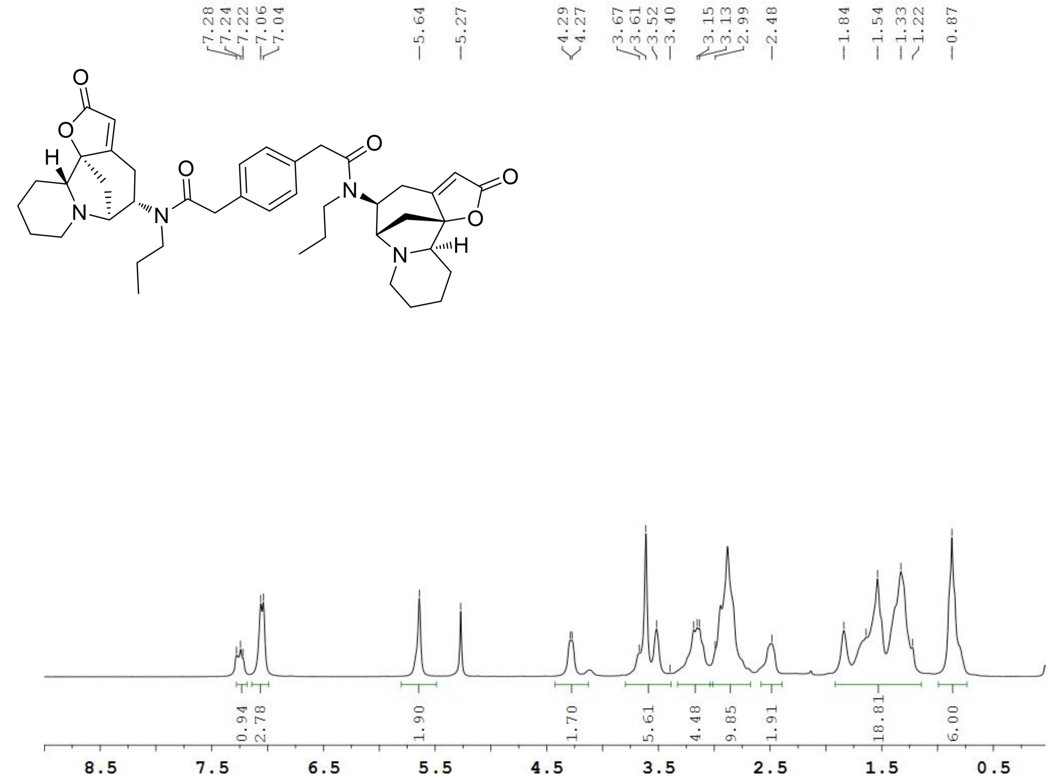

^{13}C NMR spectrum of **R4** (75 MHz, $CDCl_3$)

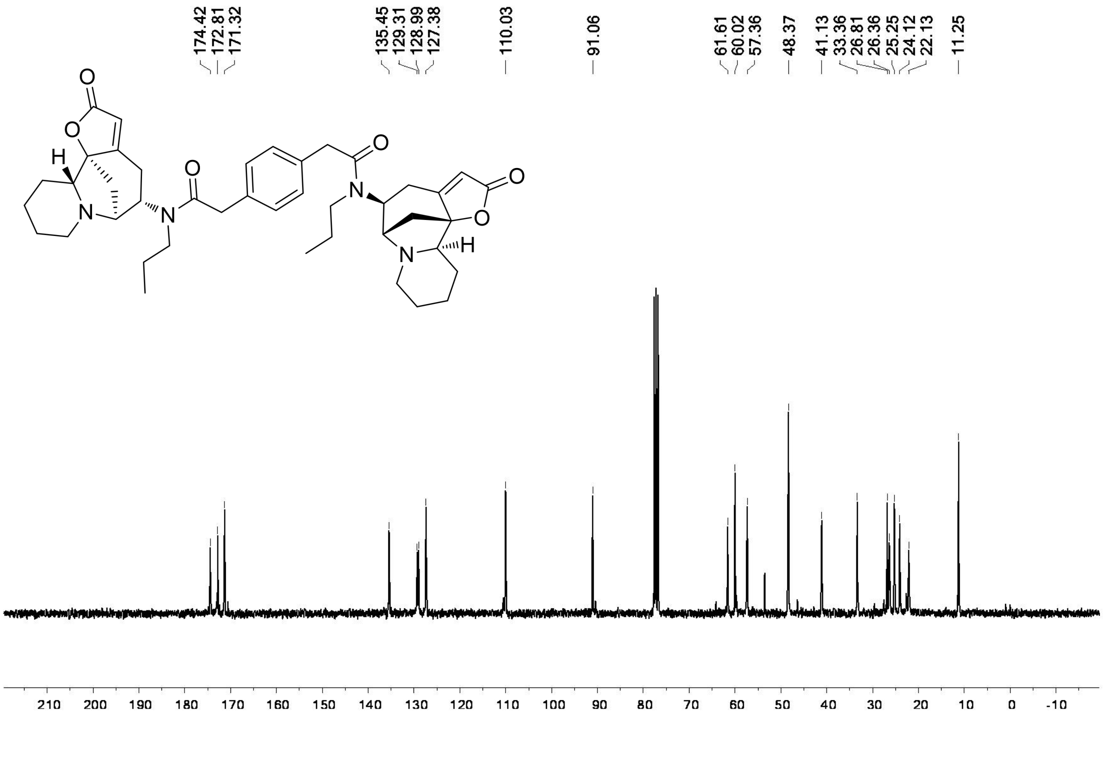

ESI-MS spectrum of **R4**

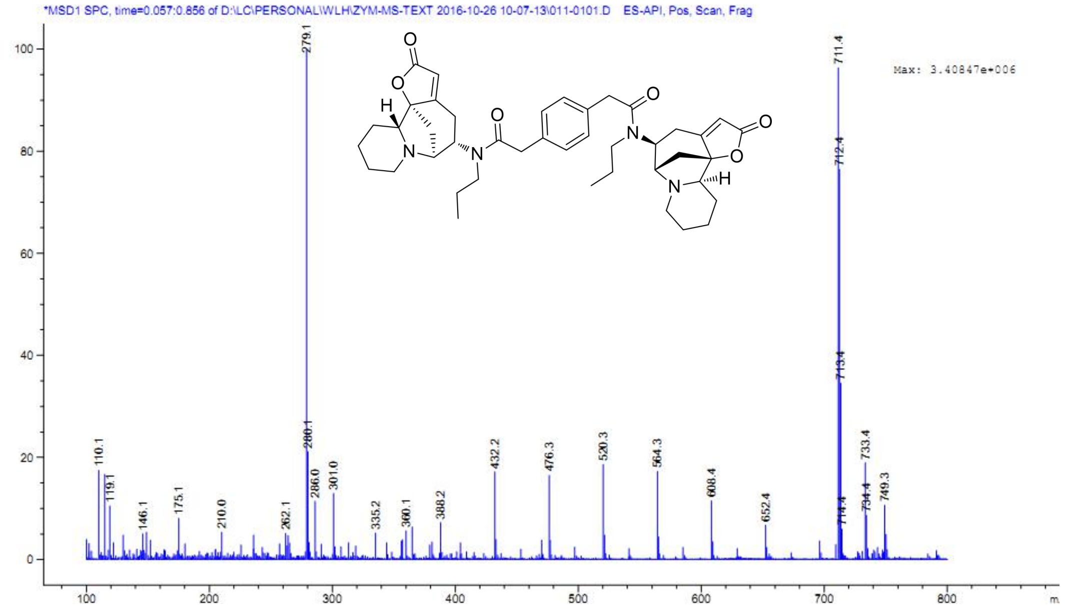

HRMS of compound **R4**

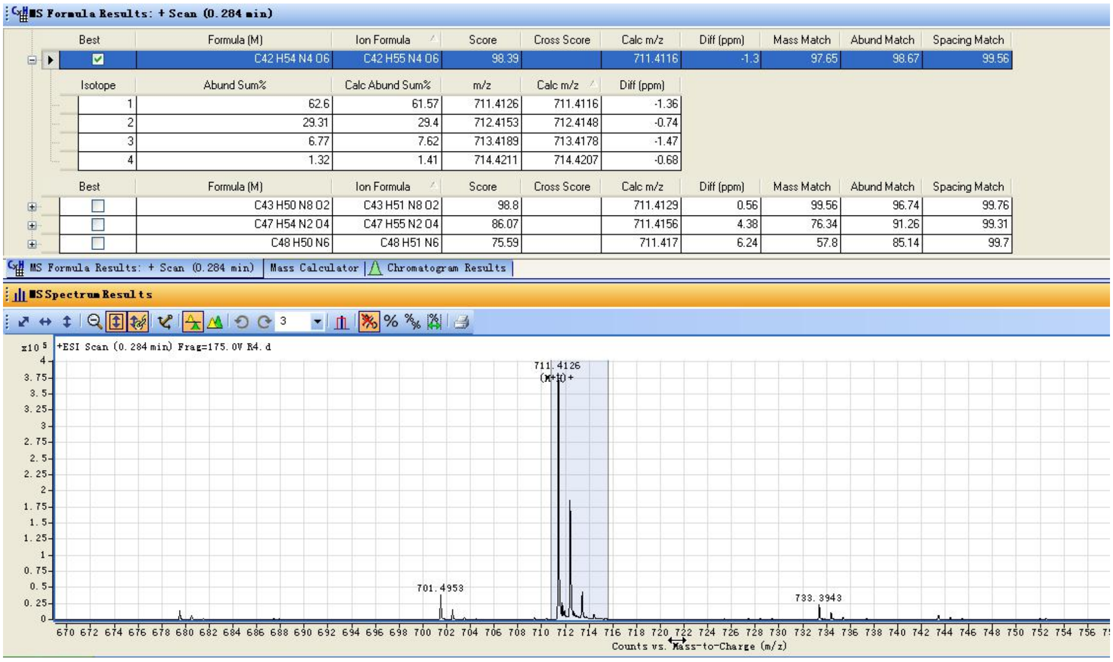

Best	Formula (M)	Ion Formula	Score	Cross Score	Calc m/z	Diff (ppm)	Mass Match	Abund Match	Spacing Match
☑	C42 H54 N4 O6	C42 H55 N4 O6	98.39		711.4116	-1.3	97.65	98.67	99.56

Isotope	Abund Sum%	Calc Abund Sum%	m/z	Calc m/z	Diff (ppm)
1	62.6	61.57	711.4126	711.4116	-1.36
2	29.31	29.4	712.4153	712.4148	-0.74
3	6.77	7.62	713.4189	713.4178	-1.47
4	1.32	1.41	714.4211	714.4207	-0.68

Best	Formula (M)	Ion Formula	Score	Cross Score	Calc m/z	Diff (ppm)	Mass Match	Abund Match	Spacing Match
☐	C43 H50 N8 O2	C43 H51 N8 O2	98.8		711.4129	0.56	99.56	96.74	99.76
☐	C47 H54 N2 O4	C47 H55 N2 O4	86.07		711.4156	4.38	76.34	91.26	99.31
☐	C48 H50 N6	C48 H51 N6	75.59		711.417	6.24	57.8	85.14	99.7

^{1}H NMR spectrum of **R5** (300 MHz, $CDCl_3$)

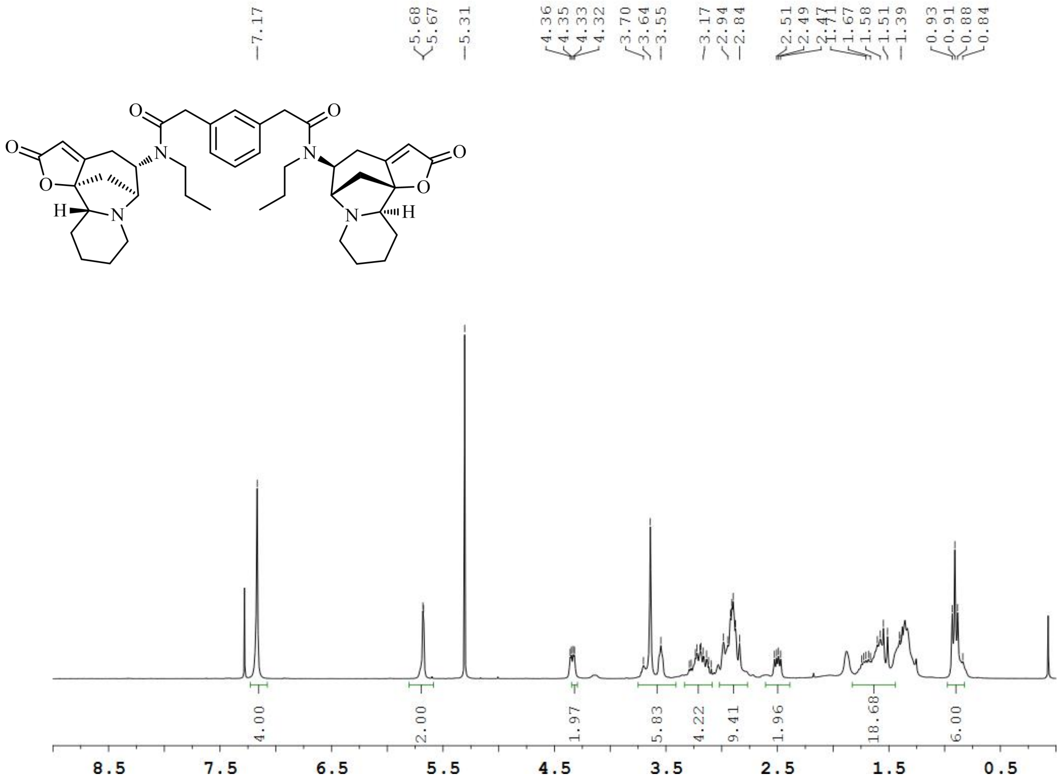

^{13}C NMR spectrum of **R5** (75 MHz, $CDCl_3$)

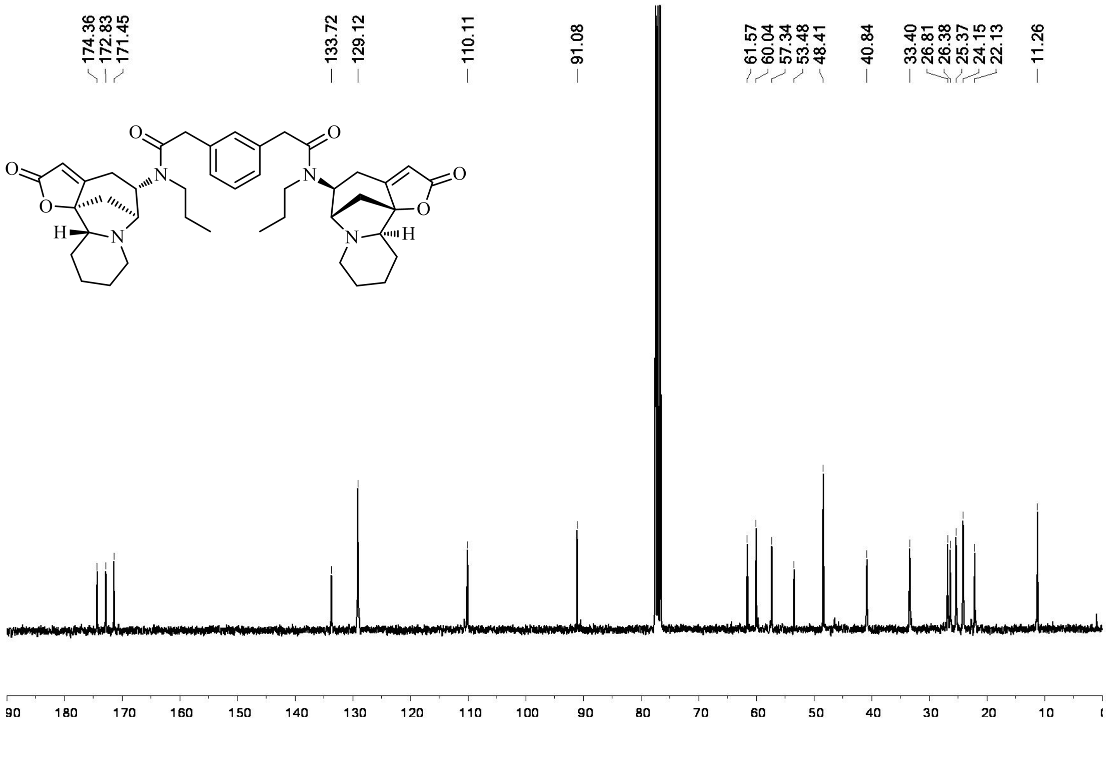

ESI-MS spectrum of **R5**

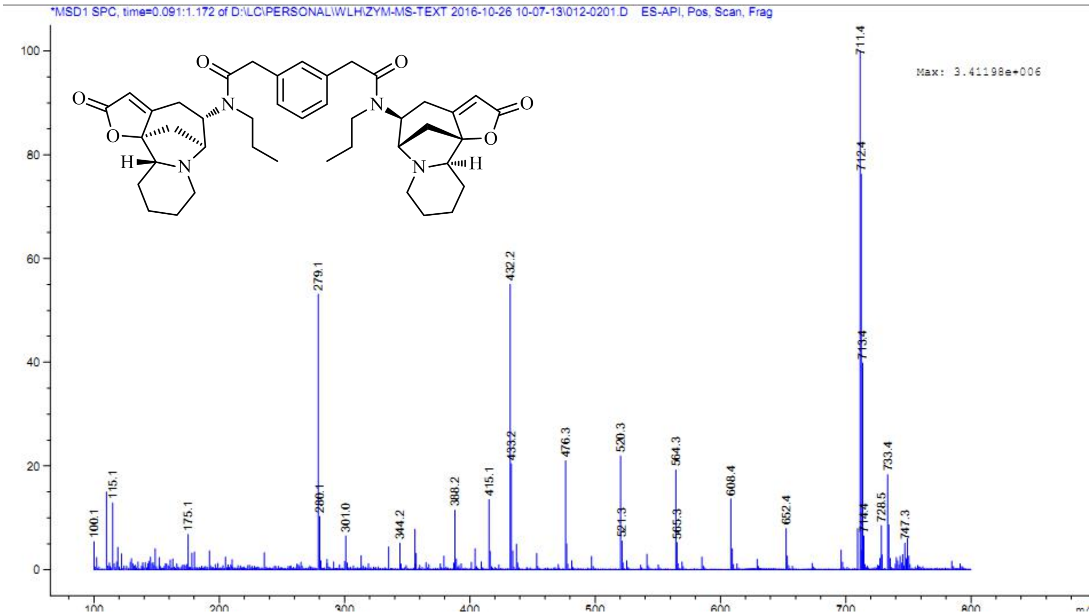

HRMS of compound **R5**

MS Formula Results: + Scan (0.284 min)

Best	Formula (M)	Ion Formula	Score	Cross Score	Calc m/z	Diff (ppm)	Mass Match	Abund Match	Spacing Match
☑	C42 H54 N4 O6	C42 H55 N4 O6	98.39		711.4116	-1.3	97.65	98.67	99.56

Isotope	Abund Sum%	Calc Abund Sum%	m/z	Calc m/z	Diff (ppm)
1	62.6	61.57	711.4126	711.4116	-1.36
2	29.31	29.4	712.4153	712.4148	-0.74
3	6.77	7.62	713.4189	713.4178	-1.47
4	1.32	1.41	714.4211	714.4207	-0.68

Best	Formula (M)	Ion Formula	Score	Cross Score	Calc m/z	Diff (ppm)	Mass Match	Abund Match	Spacing Match
☐	C43 H50 N8 O2	C43 H51 N8 O2	98.8		711.4129	0.56	99.56	96.74	99.76
☐	C47 H54 N2 O4	C47 H55 N2 O4	86.07		711.4156	4.38	76.34	91.26	99.31
☐	C48 H50 N6	C48 H51 N6	75.59		711.417	6.24	57.8	85.14	99.7

MS Formula Results: + Scan (0.284 min) | Mass Calculator | Chromatogram Results

MS Spectrum Results

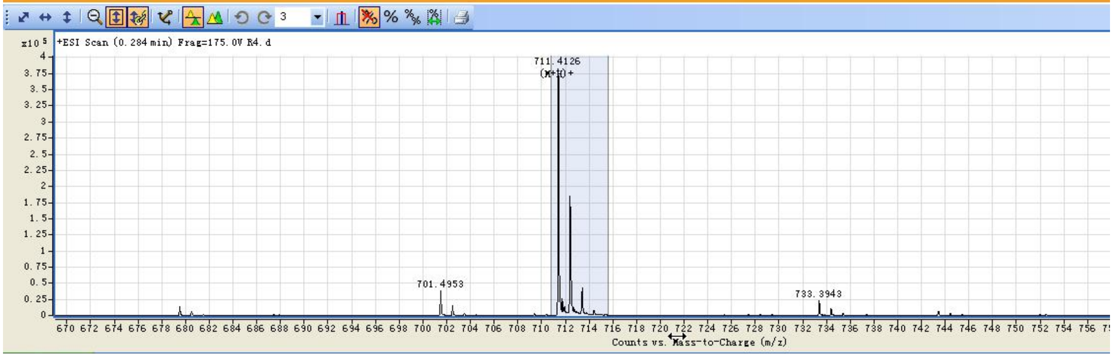

^{1}H NMR spectrum of **R6** (300 MHz, $CDCl_3$)

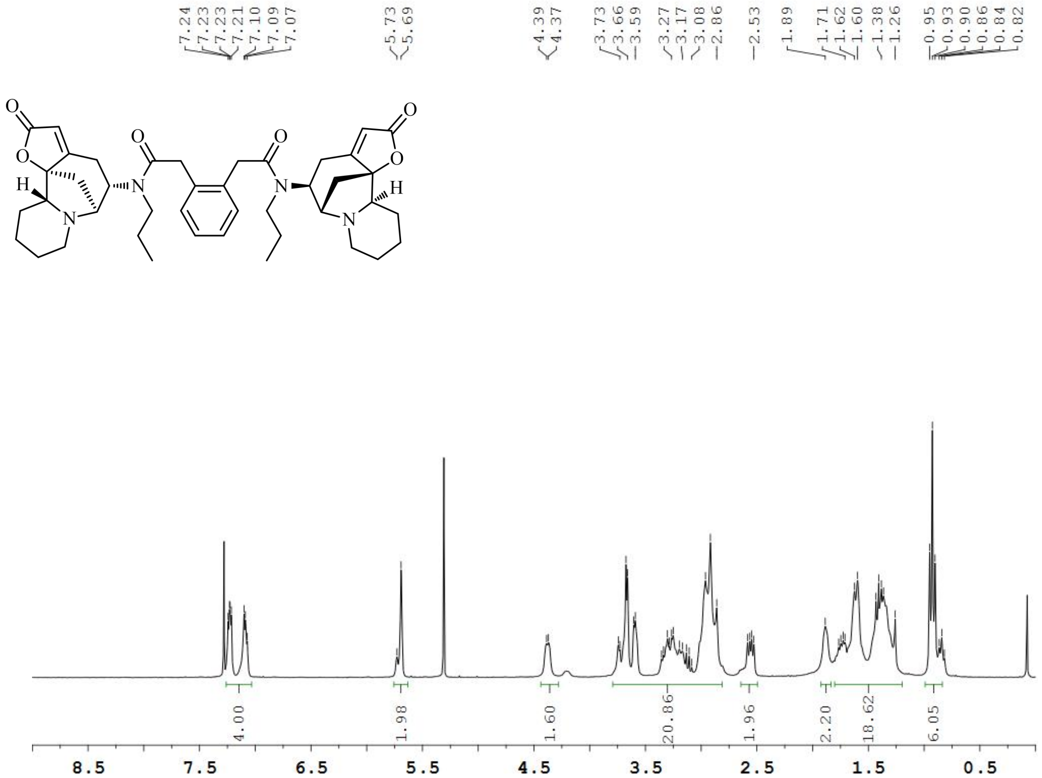

^{13}C NMR spectrum of **R6** (75 MHz, $CDCl_3$)

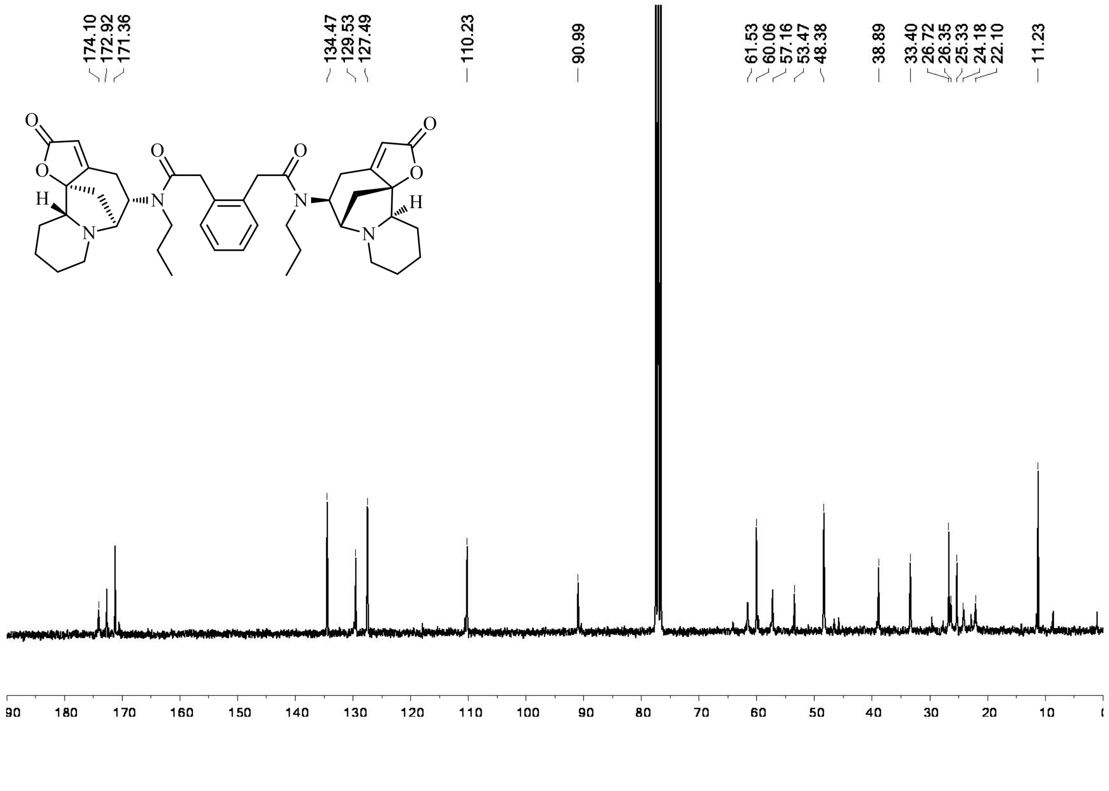

ESI-MS spectrum of **R6**

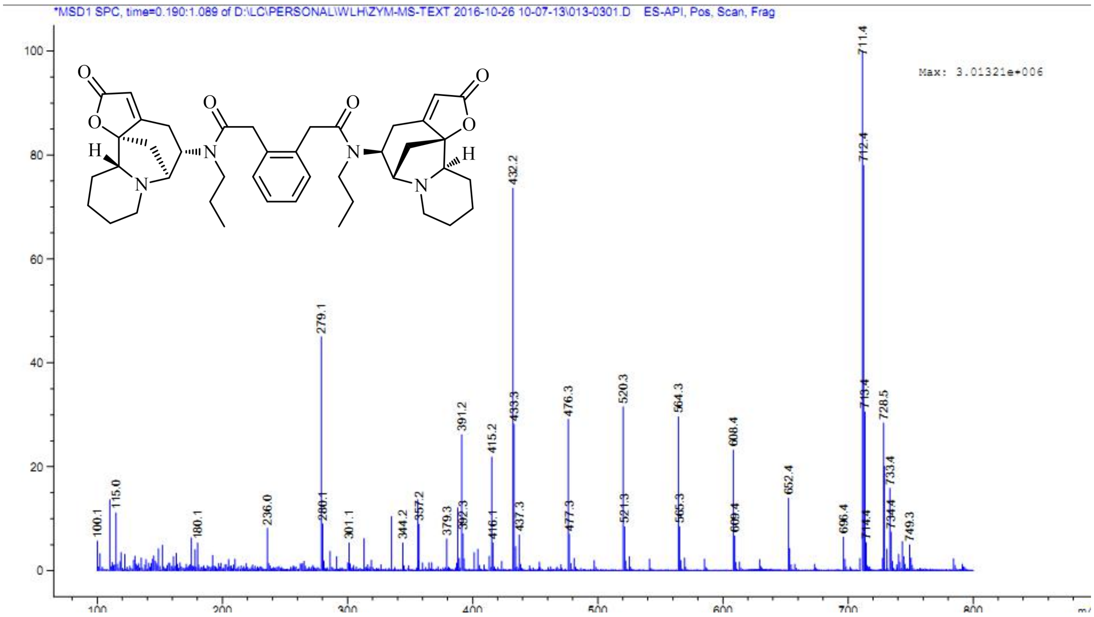

HRMS of compound **R6**

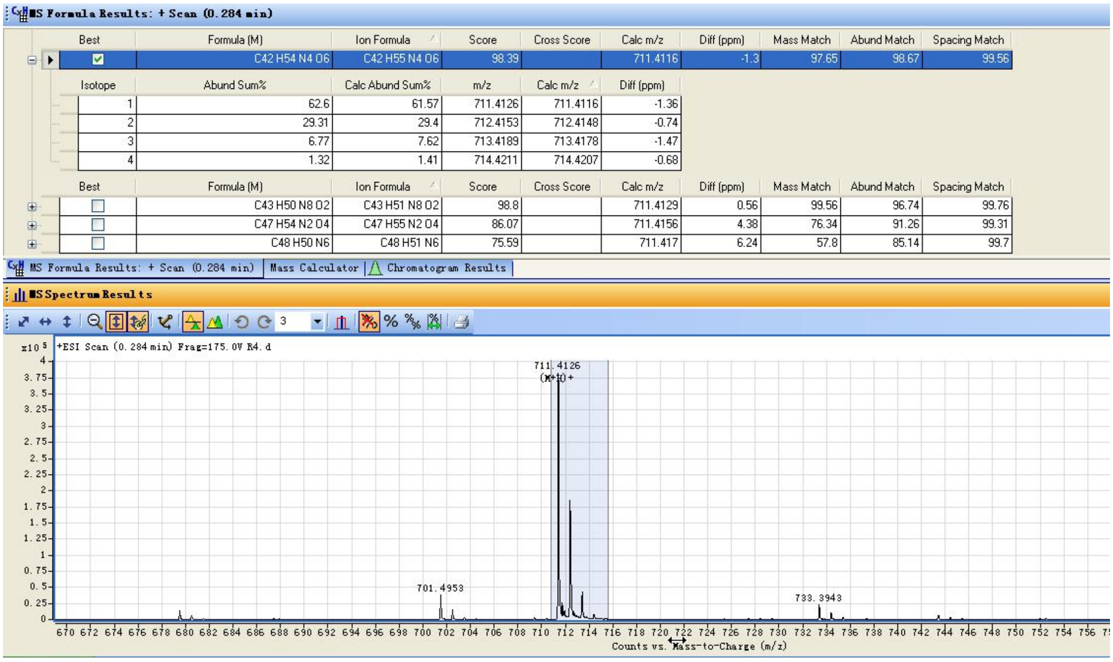

Best	Formula (M)	Ion Formula	Score	Cross Score	Calc m/z	Diff (ppm)	Mass Match	Abund Match	Spacing Match
☑	C42 H54 N4 O6	C42 H55 N4 O6	98.39		711.4116	-1.3	97.65	98.67	99.56

Isotope	Abund Sum%	Calc Abund Sum%	m/z	Calc m/z	Diff (ppm)
1	62.6	61.57	711.4126	711.4116	-1.36
2	29.31	29.4	712.4153	712.4148	-0.74
3	6.77	7.62	713.4189	713.4178	-1.47
4	1.32	1.41	714.4211	714.4207	-0.68

Best	Formula (M)	Ion Formula	Score	Cross Score	Calc m/z	Diff (ppm)	Mass Match	Abund Match	Spacing Match
☐	C43 H50 N8 O2	C43 H51 N8 O2	98.8		711.4129	0.56	99.56	96.74	99.76
☐	C47 H54 N2 O4	C47 H55 N2 O4	86.07		711.4156	4.38	76.34	91.26	99.31
☐	C48 H50 N6	C48 H51 N6	75.59		711.417	6.24	57.8	85.14	99.7

^{1}H NMR spectrum of **HR1** (300 MHz, $CDCl_3$)

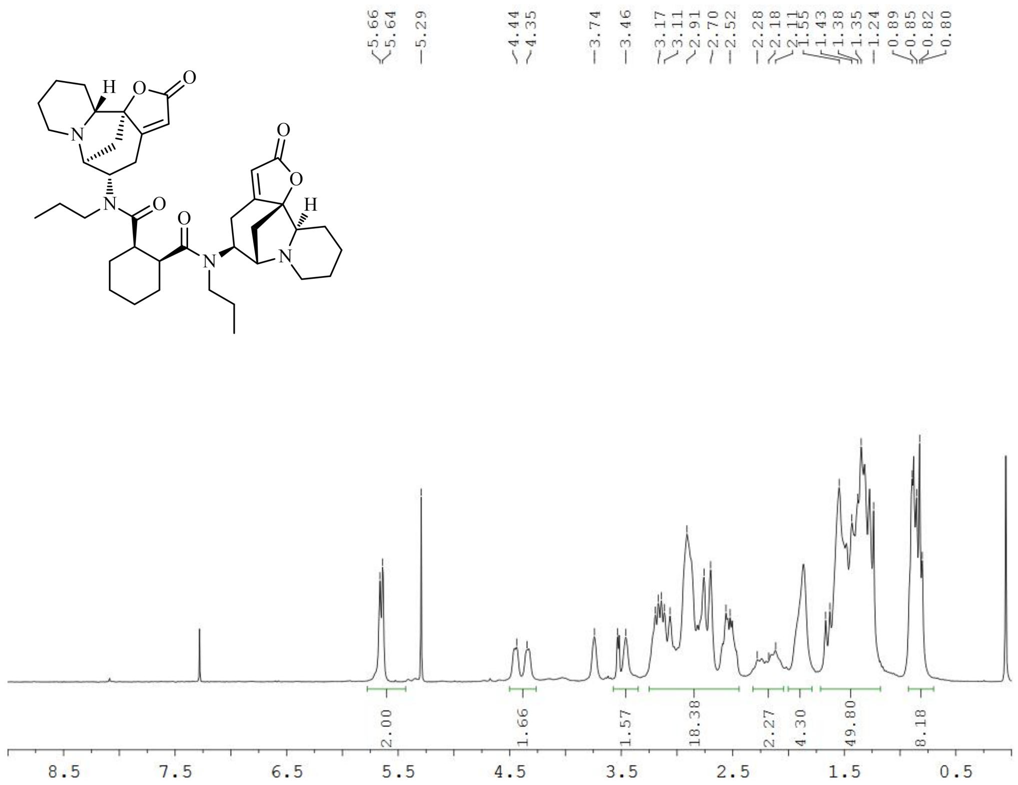

^{13}C NMR spectrum of **HR1** (75 MHz, $CDCl_3$)

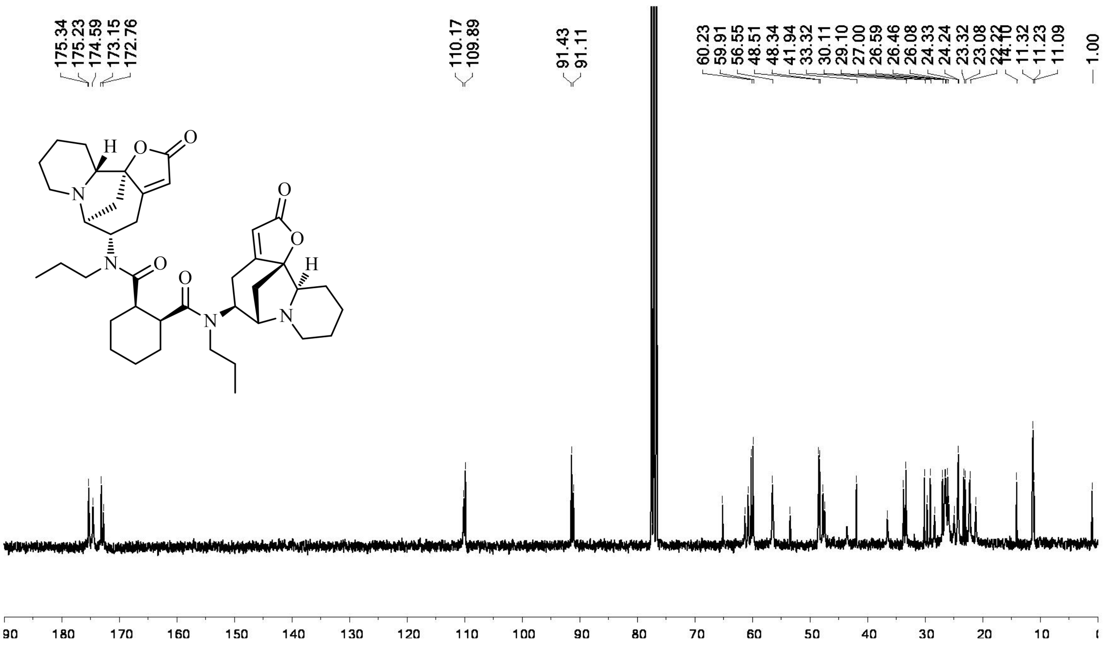

ESI-MS spectrum of **HR1**

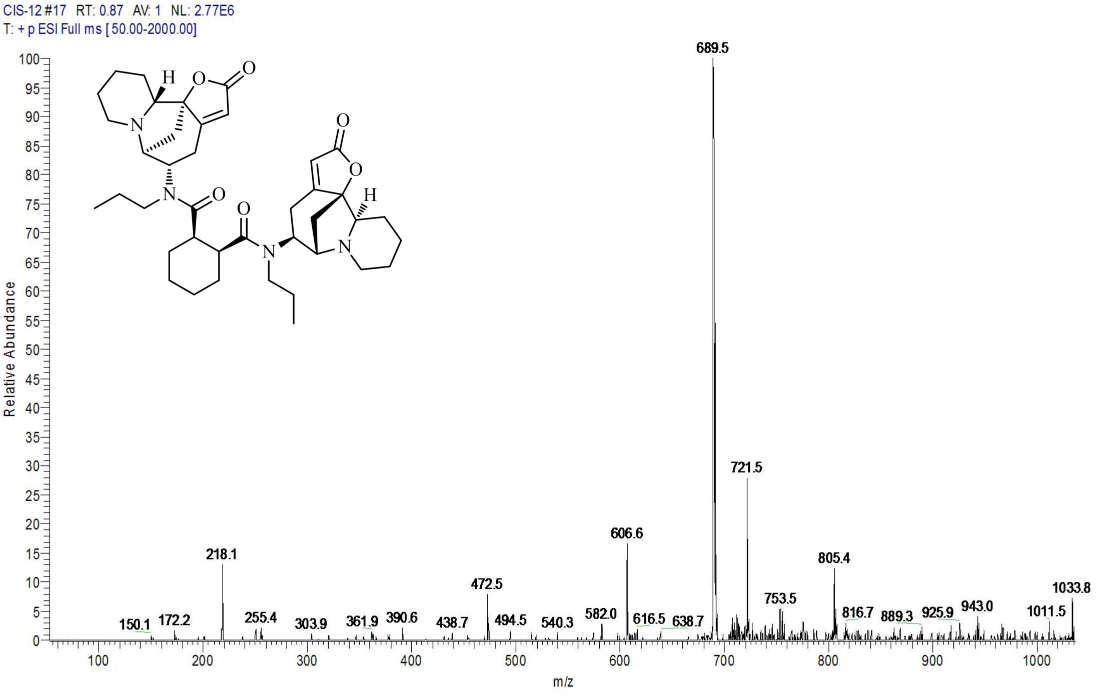

HRMS of compound **HR1**

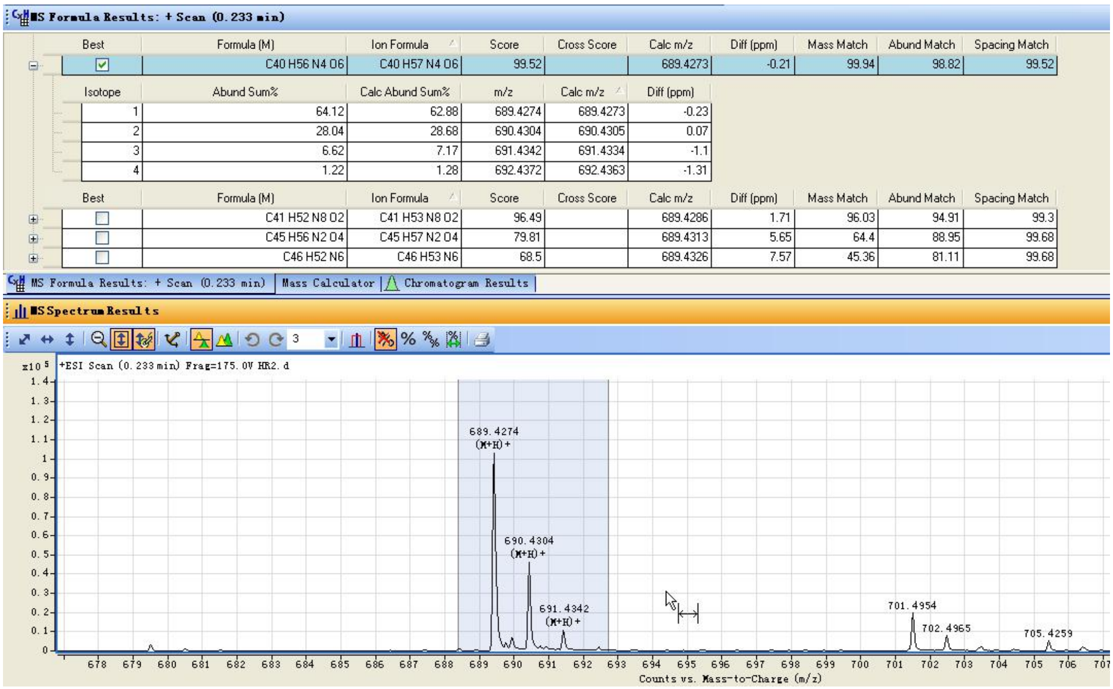

MS Formula Results: + Scan (0.233 min)

Best	Formula (M)	Ion Formula	Score	Cross Score	Calc m/z	Diff (ppm)	Mass Match	Abund Match	Spacing Match
☑	C40 H56 N4 O6	C40 H57 N4 O6	99.52		689.4273	-0.21	99.94	98.82	99.52

Isotope	Abund Sum%	Calc Abund Sum%	m/z	Calc m/z	Diff (ppm)
1	64.12	62.88	689.4274	689.4273	-0.23
2	28.04	28.68	690.4304	690.4305	0.07
3	6.62	7.17	691.4342	691.4334	-1.1
4	1.22	1.28	692.4372	692.4363	-1.31

Best	Formula (M)	Ion Formula	Score	Cross Score	Calc m/z	Diff (ppm)	Mass Match	Abund Match	Spacing Match
☐	C41 H52 N8 O2	C41 H53 N8 O2	96.49		689.4286	1.71	96.03	94.91	99.3
☐	C45 H56 N2 O4	C45 H57 N2 O4	79.81		689.4313	5.65	64.4	88.95	99.68
☐	C46 H52 N6	C46 H53 N6	68.5		689.4326	7.57	45.36	81.11	99.68

^{1}H NMR spectrum of **HR2** (300 MHz, $CDCl_3$)

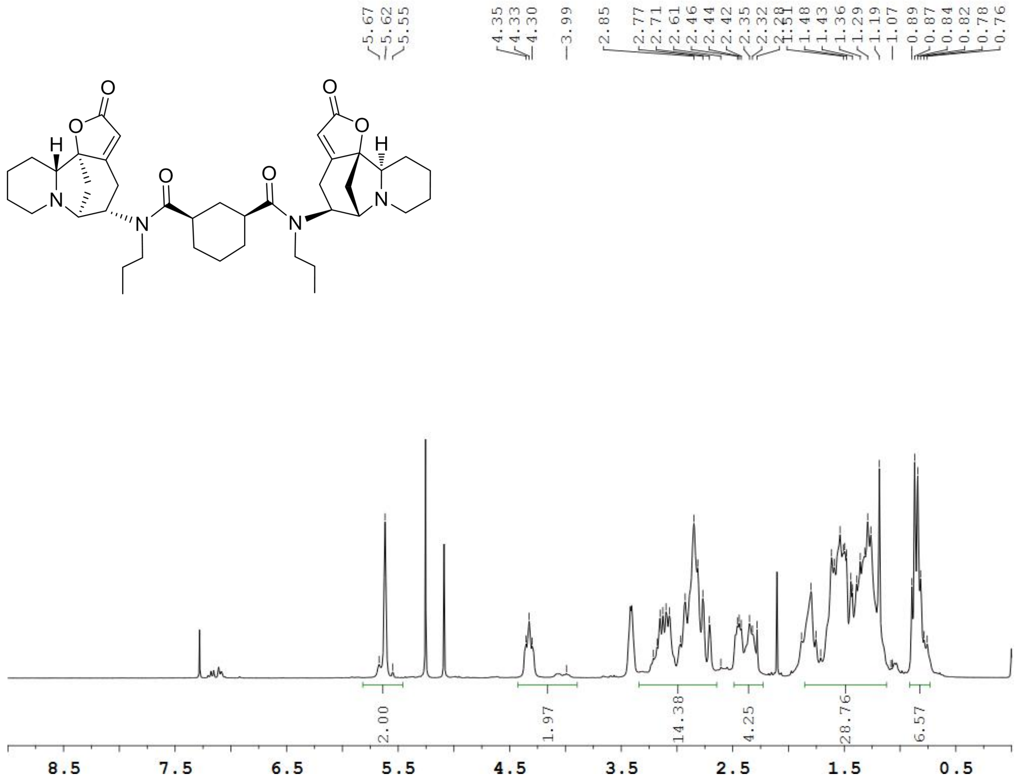

^{13}C NMR spectrum of **HR2** (75 MHz, $CDCl_3$)

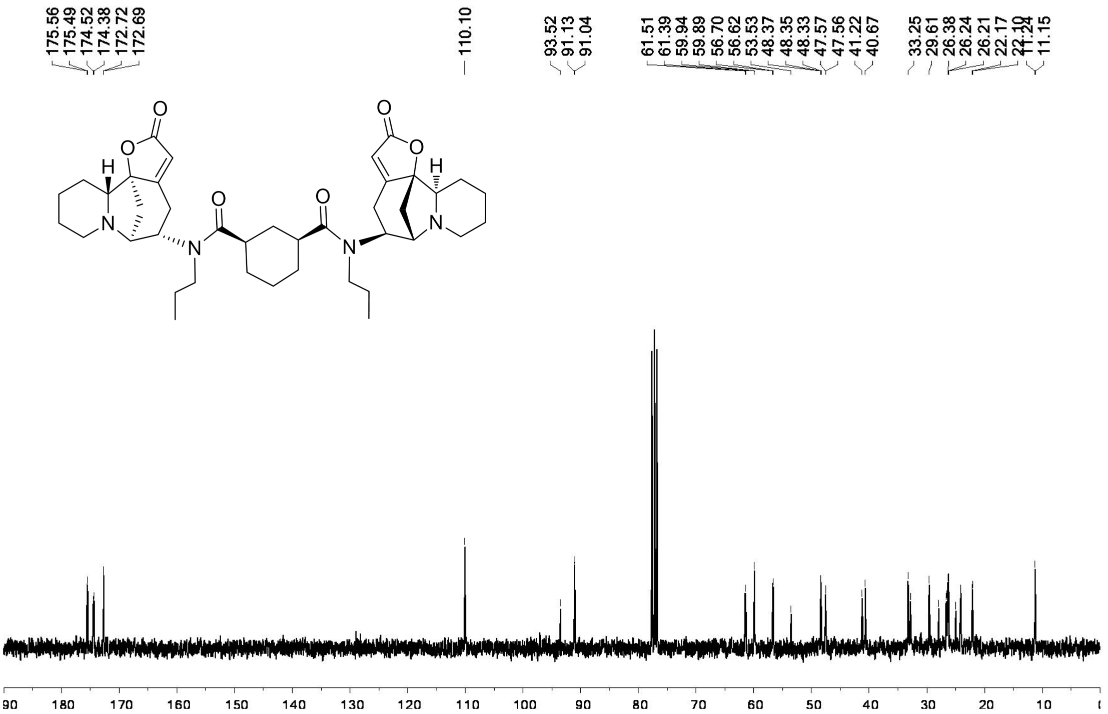

ESI-MS spectrum of **HR2**

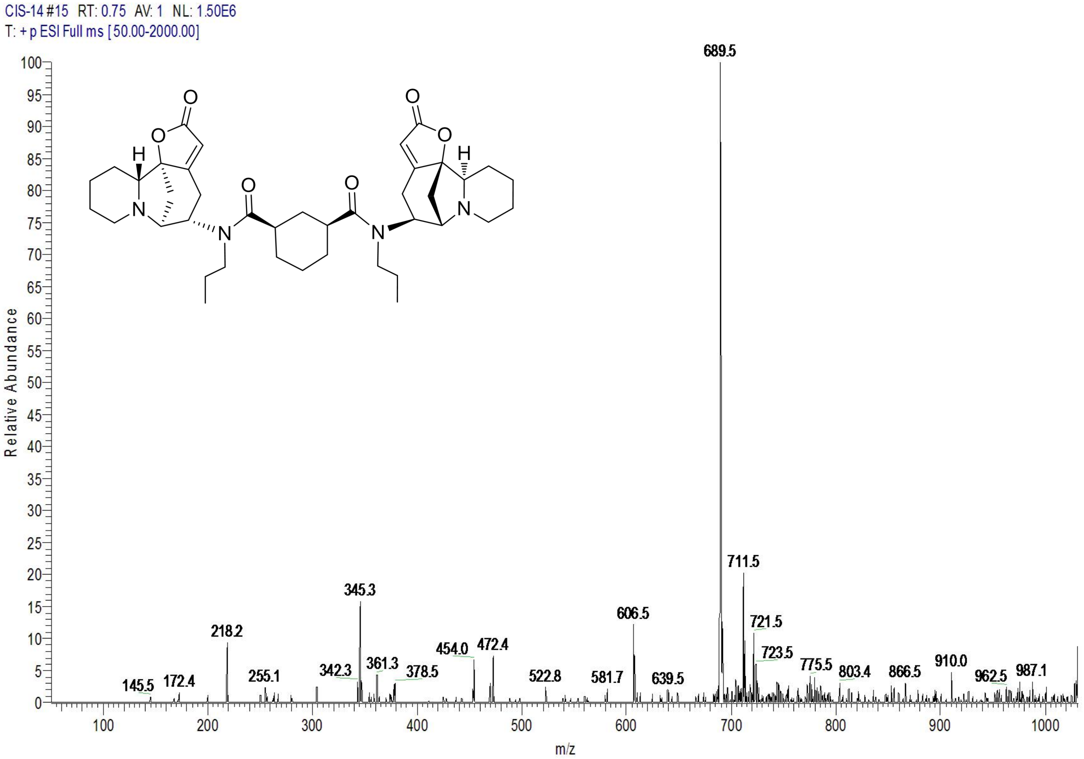

HRMS of compound **HR2**

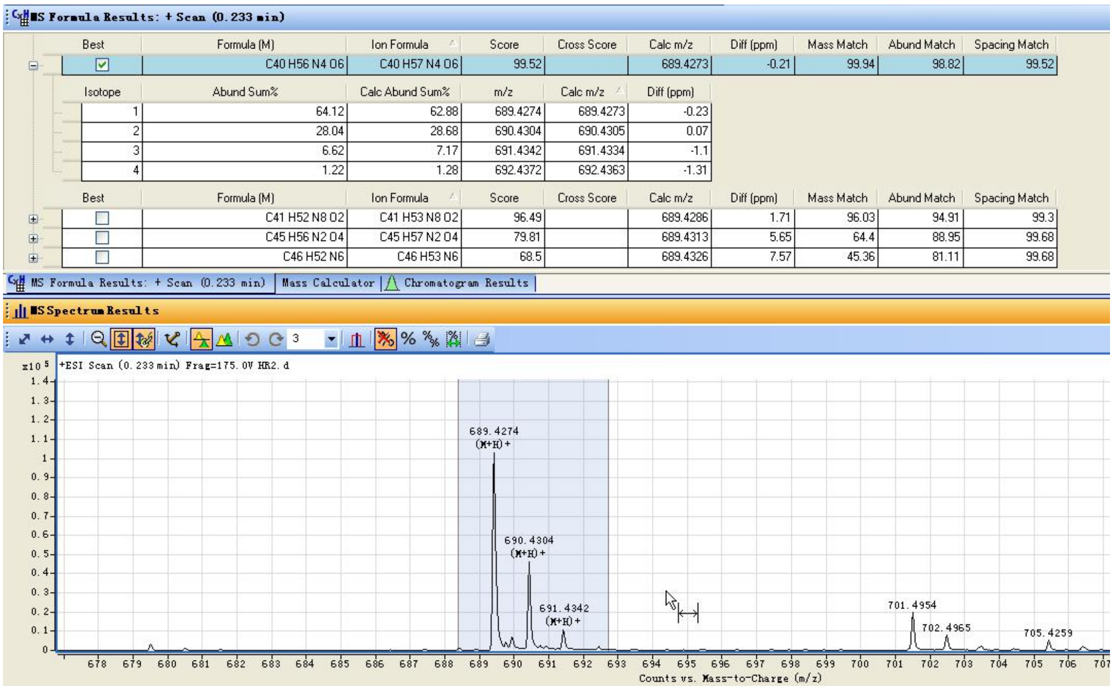

MS Formula Results: + Scan (0.233 min)

Best	Formula (M)	Ion Formula	Score	Cross Score	Calc m/z	Diff (ppm)	Mass Match	Abund Match	Spacing Match
☑	C40 H56 N4 O6	C40 H57 N4 O6	99.52		689.4273	-0.21	99.94	98.82	99.52

Isotope	Abund Sum%	Calc Abund Sum%	m/z	Calc m/z	Diff (ppm)
1	64.12	62.88	689.4274	689.4273	-0.23
2	28.04	28.68	690.4304	690.4305	0.07
3	6.62	7.17	691.4342	691.4334	-1.1
4	1.22	1.28	692.4372	692.4363	-1.31

Best	Formula (M)	Ion Formula	Score	Cross Score	Calc m/z	Diff (ppm)	Mass Match	Abund Match	Spacing Match
☐	C41 H52 N8 O2	C41 H53 N8 O2	96.49		689.4286	1.71	96.03	94.91	99.3
☐	C45 H56 N2 O4	C45 H57 N2 O4	79.81		689.4313	5.65	64.4	88.95	99.68
☐	C46 H52 N6	C46 H53 N6	68.5		689.4326	7.57	45.36	81.11	99.68

^{1}H NMR spectrum of **HR3** (300 MHz, $CDCl_3$)

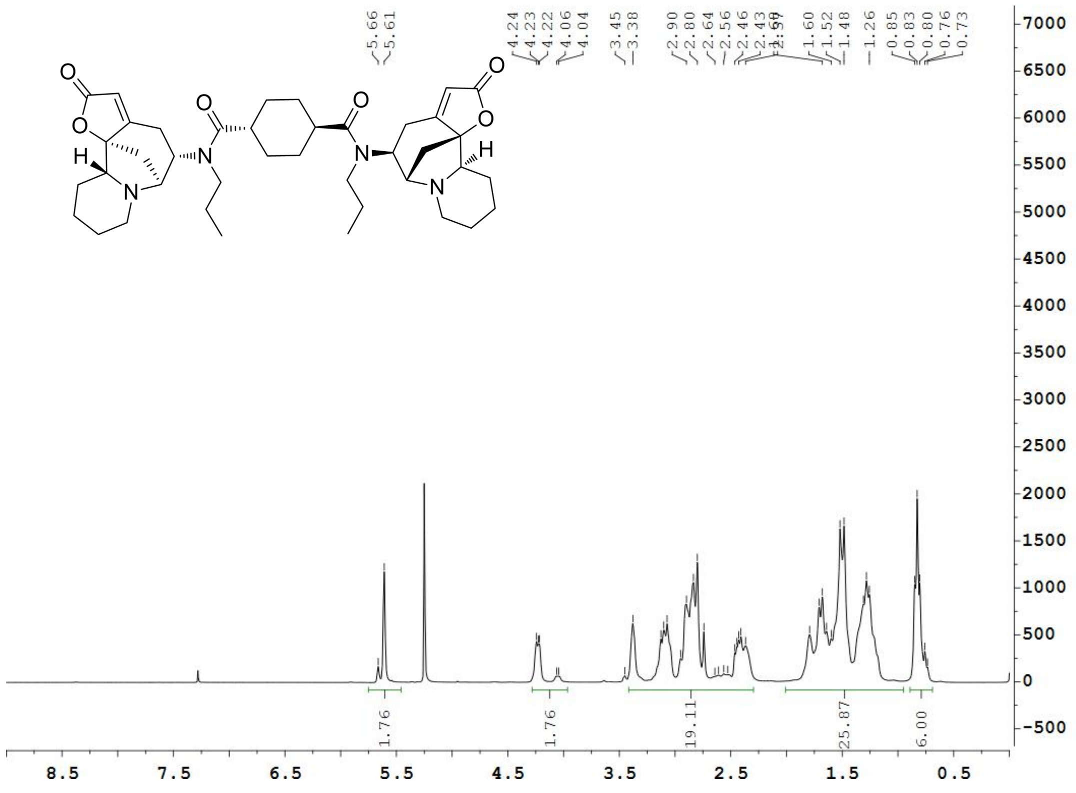

^{13}C NMR spectrum of **HR3** (75 MHz, $CDCl_3$)

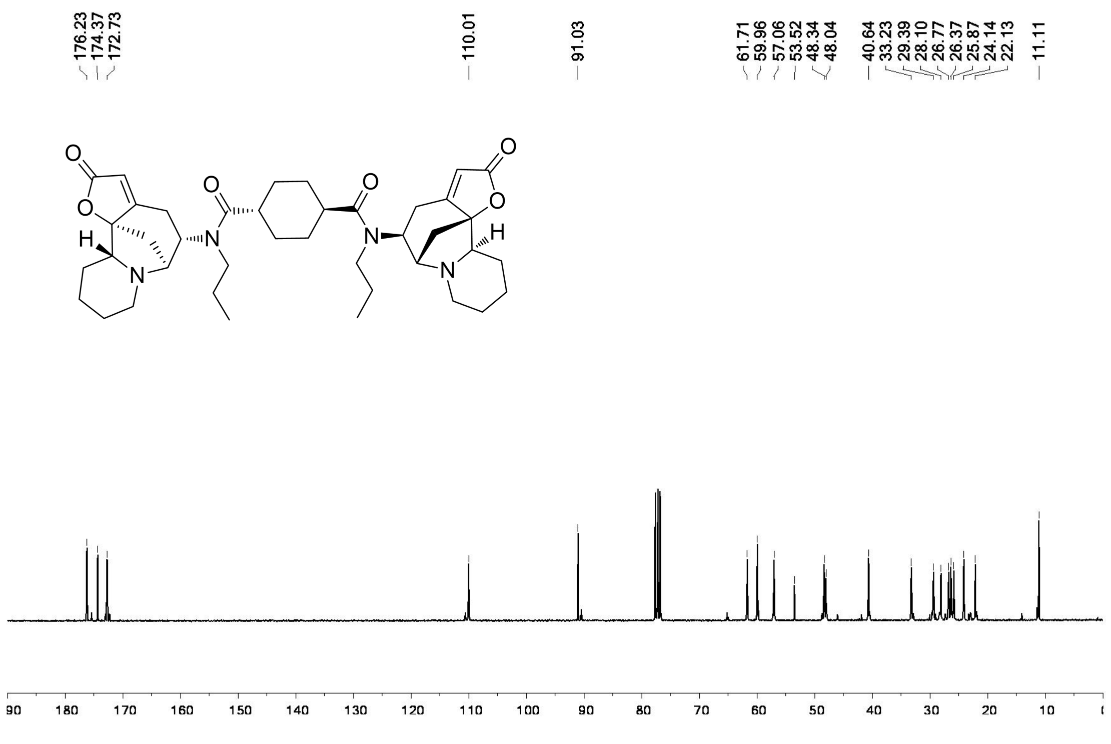

ESI-MS spectrum of **HR3**

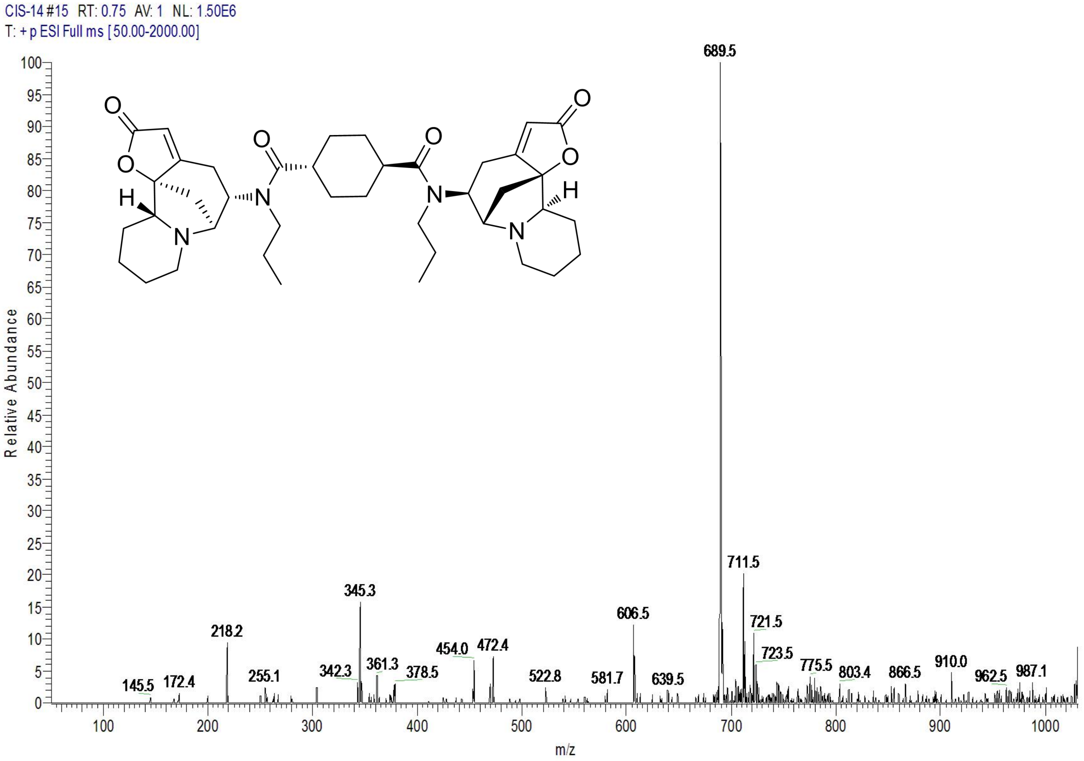

HRMS of compound **HR3**

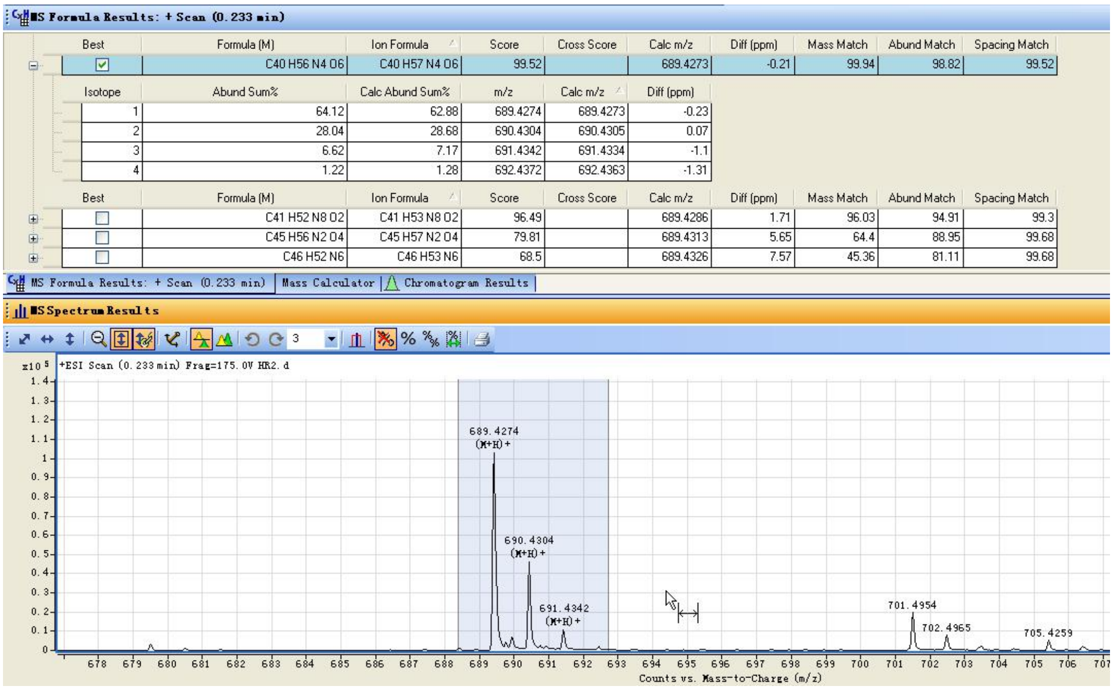

MS Formula Results: + Scan (0.233 min)

Best	Formula (M)	Ion Formula	Score	Cross Score	Calc m/z	Diff (ppm)	Mass Match	Abund Match	Spacing Match
☑	C40 H56 N4 O6	C40 H57 N4 O6	99.52		689.4273	-0.21	99.94	98.82	99.52

Isotope	Abund Sum%	Calc Abund Sum%	m/z	Calc m/z	Diff (ppm)
1	64.12	62.88	689.4274	689.4273	-0.23
2	28.04	28.68	690.4304	690.4305	0.07
3	6.62	7.17	691.4342	691.4334	-1.1
4	1.22	1.28	692.4372	692.4363	-1.31

Best	Formula (M)	Ion Formula	Score	Cross Score	Calc m/z	Diff (ppm)	Mass Match	Abund Match	Spacing Match
☐	C41 H52 N8 O2	C41 H53 N8 O2	96.49		689.4286	1.71	96.03	94.91	99.3
☐	C45 H56 N2 O4	C45 H57 N2 O4	79.81		689.4313	5.65	64.4	88.95	99.68
☐	C46 H52 N6	C46 H53 N6	68.5		689.4326	7.57	45.36	81.11	99.68
